# Non-Hermitian topology in static mechanical metamaterials

**DOI:** 10.1126/sciadv.adf7299

**Published:** 2023-07-05

**Authors:** Aoxi Wang, Zhiqiang Meng, Chang Qing Chen

**Affiliations:** Department of Engineering Mechanics, CNMM and AML, Tsinghua University, Beijing 100084, PR China.

## Abstract

The combination of broken Hermiticity and band topology in physical systems unveils a novel bound state dubbed as the non-Hermitian skin effect (NHSE). Active control that breaks reciprocity is usually used to achieve NHSE, and gain and loss in energy are inevitably involved. Here, we demonstrate non-Hermitian topology in a mechanical metamaterial system by exploring its static deformation. Nonreciprocity is introduced via passive modulation of the lattice configuration without resorting to active control and energy gain/loss. Intriguing physics such as the reciprocal and higher-order skin effects can be tailored in the passive system. Our study provides an easy-to-implement platform for the exploration of non-Hermitian and nonreciprocal phenomena beyond conventional wave dynamics.

## Teaser

Non-Hermitian topology is tailored in static mechanical systems without resorting to active control.

## INTRODUCTION

Non-Hermiticity is extensively accepted in describing nonequilibrium or open dynamic systems that exchange their energy with the environment (*1*, *2*). Experimental progress in optics markedly stimulates the development of non-Hermitian physics, whereby gain and loss are ubiquitous and dynamic evolution of the optical waves can be characterized by non-Hermitian Hamiltonians (*3*–*6*), which has already been extended to other physical systems with controlled gain/loss effect (*7*–*13*). Reciprocity is a fundamental principle in many physical realms, such as the helical edge state in 2D topological insulators with time-reversal symmetry (TRS) protection (*14*) and Lorentz reciprocity in electromagnetism (*15*). Reciprocity in mechanical systems is embedded with dual implications: One is derived from Newton’s third law, and the other is the so-called Maxwell-Betti reciprocity (*16*). Although the former is a footstone in classical mechanics and holds in many physical images, the latter is unique for linear systems concerning the symmetry between perturbation and response (*17*) and can be broken by either active modulation (*18*–*20*) or in the presence of nonlinearity (*21*, *22*).

The combination of non-Hermiticity and topological band theory not only enriches the conventional eigenstate-based band topology, such as the gain/loss-induced nontrivial topological phase (*23*) and the photonic zero mode induced by the parity-time (PT) symmetry phase transition (*24*), but also discloses a novel class of topological phases that is characterized by the spectral winding topology (*25*–*29*). Probably the most intriguing phenomenon of the latter spectral topology is the non-Hermitian skin effect (NHSE) where all macroscopic bulk wave functions are squeezed toward the boundary for a finite-sized system (*30*). Breaking reciprocity and generating a biased directional flow are necessary to pump a particle to the boundary and induce the NHSE (*31*). This is in general nontrivial and is usually fulfilled via active modulation of system parameters (*18*) that inevitably injects/extracts energy into/from the closed system. Here, focusing on the static deformation pattern in mechanical metamaterials, we map the wave dynamics to a static system by concretizing the time *t* to a space dimension *n* accompanied with an imaginary transform, *n* = *it*. Crucially, this enables us to tailor an effective nonreciprocity by passively adjusting the metamaterial configurations without external/active actuation. Consequently, exotic topological effects, such as the reciprocal skin effect and higher-order skin effect that are exclusive to open dynamic systems (*26*, *32*–*34*), can be observed in our conservative, passive static system.

## RESULTS

### Correspondence between static Rayleigh mode in planar lattices and one-dimensional wave dynamics

**Fig. 1.**
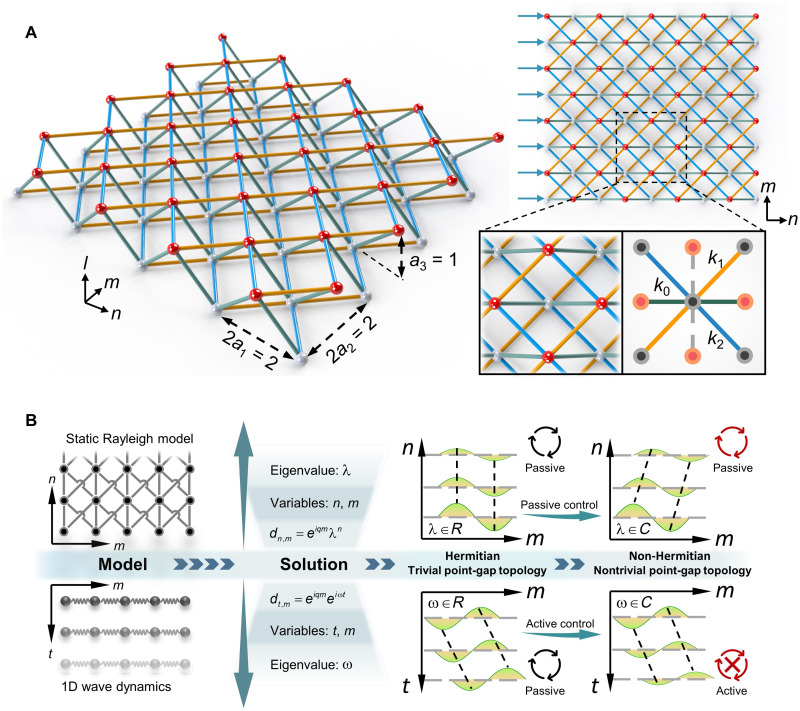
Static nonreciprocal metamaterial and its dynamic counterparts. (**A**) Schematic of the static nonreciprocal metamaterial, where stiffnesses of bars between adjacent nodes are *k_i_*, with *i* = 0,1,2 denoting different directions, and the load is applied on the left boundary. Left and top right panels are side and top views of the metamaterial, respectively, while the associate cell is drawn at the bottom right panel. (**B**) Illustration of the mathematical equivalence between static Rayleigh model and 1D wave dynamics, where the time coordinate in dynamics is replaced by an additional space coordinate in our static model.

The mechanical metamaterial considered in this study is a lattice structure extending along the *n* and *m* axes and comprising two sets of nodes (red and gray spheres) periodically distributed in two adjacent layers along the *l* axis, with colored bars representing linear interactions between adjacent nodes (Fig. 1A). The bars can be either stretch-dominated truss or bending-dominated frame. The lattice constants (*a*_1_, *a*_2_, *a*_3_) along three axes are normalized for convenience, and nodes are labeled by a pair of planar indices, (*n*, *m*), according to their projection on the *n*−*m* plane. Only translation displacement in the *n* direction is allowed for all nodes. The lattice stiffnesses of the horizontal bars (olive bars) and the two diagonal bars (aurantia and cyanine bars) are denoted by *k*_0_, *k*_1_, and *k*_2_, respectively, while the coupling along the *m* direction is neglected (gray dashed lines). Here, the stiffness of a bar is defined by the corresponding force for a given unit relative displacement between two end nodes. External loads are applied on the left edge (*n* = 0) and the lattice extends infinitely along the *n* direction (i.e., *n*→∞).

For a prescribed static Bloch form displacement on the boundary *n* = 0, i.e., *d*_0,*m*_ = *ce^iqm^*, the resulted displacement field within the bulk is *d*_*n*,*m*_ = *ce^iqm^e*^−η*n*^ with *q* and η denoting the wave number and decay factor, respectively. This deformation mode is referred to as the static Rayleigh deformation mode in this study by noting that it resembles the motion of surface Rayleigh waves (*35*), i.e., both share a similar feature of exponential attenuation of sinusoidal boundary displacement into bulk. Under the periodic boundary condition (PBC) in the *m* direction, the quasistatic equilibrium equation for inner nodes (0 < *n* < ∞) is given by (see Materials and Methods for details)

$$\begin{array}{c} -(\mu k_{0}+\mu k_{1}+\mu k_{2}-k_{t})d_{0,m}=(e^{-\eta }k_{1}+e^{\eta }k_{2})(d_{0,m+1}-d_{0,m})\\ +(e^{-\eta }k_{2}+e^{\eta }k_{1})(d_{0,m-1}-d_{0,m}) \end{array}$$ (1)

where *k*_t_ = 2(*k*_0_ + *k*_1_ + *k*_2_) represents the total stiffness of the associate cell characterizing the minimal unit including all interactions related to node (*n*, *m*) (Fig. 1A) and μ = *e*^−η^ + *e*^η^. The above formula is mathematically equivalent to the dynamic equation of the one-dimensional (1D) mass-spring model, in which the coupling stiffnesses in opposite directions are denoted by *k*_L→R_ and *k*_R→L_, respectively. Reciprocity is maintained when *k*_L→R_ = *k*_R→L_, whereas the nonreciprocity (*k*_L→R_ ≠ *k*_R→L_) can be involved by external actuation (*18*). From Eq. 1, we can see that the effective stiffnesses in opposite directions, i.e., *m*→*m* + 1 and *m* + 1→*m*, can be formally expressed as *k*_*m*→*m*+1_ = *e*^−η^*k*_2_ + *e*^η^*k*_1_ and *k*_*m*+1→*m*_ = *e*^−η^*k*_1_ + *e*^η^*k*_2_, respectively. Analogous to its dynamic counterpart, reciprocity holds for symmetric configurations with *k*_1_ = *k*_2_, while nonreciprocity occurs when the symmetry is broken (i.e., *k*_1_ ≠ *k*_2_) and the static Rayleigh mode attenuates (η ≠ 0). The latter is a natural requirement of the Saint-Venant’s principle (*36*). The quantum mechanics counterpart of the above classical models is the clean Hatano-Nelson model (*37*, *38*), i.e., a 1D monoatomic tight-binding model with asymmetric hopping amplitudes. Breaking the reciprocity in these dynamic systems can be tailored by active control, e.g., via the piezoelectric effect (*16*) and the laser-induced dissipation (*8*). By contrast, nonreciprocity is achieved in our static system by passively adjusting the bar stiffness (i.e., *k*_1_ ≠ *k*_2_) without any external modulation or nonlinear components. The reason for the linear nonreciprocity in our model can be attributed to the conserved quantity of the state vectors, which, instead of being their norm as in wave dynamics, is the phase factor (see Materials and Methods). The lack of conservation of phase factor has no effects on energy flow when non-Hermiticity is invoked in our model. This is quite natural because the selected displacement field (*d*_*n*,*m*_ = *ce^iqm^e*^−η*n*^) mimics a 1D wave motion [*d_m_*(*t*) = *ce^iqm^e*^−*i*ω*t*^] via an “imaginary time,” *n* = *it*, which exchanges the identities between the amplitude amplification/attenuation and phase accumulation. The imaginary time may be invoked in a real wave system via a negative mass density or negative bulk modulus, and this mapping is an enrichment of the synthetic dimension (*39*–*41*) that usually bears a linear relation between the variables. Consequently, adding/removing additional energy to the closed system is no longer indispensable for the induced non-Hermiticity with a complex decay factor (Fig. 1B and movie S1).

By virtue of the Fourier transform, Eq. 1 can be rewritten as the following eigen-equation in the momentum space (see Materials and Methods)

$$e^{-\eta (q)}\left[\begin{array}{c} 1\\ e^{-\eta (q)} \end{array}\right]=H(q)\left[\begin{array}{c} 1\\ e^{-\eta (q)} \end{array}\right]\;H(q)=\left[\begin{array}{cc} 0 & 1\\ -\frac{k_{0}+k_{1}e^{-iq}+k_{2}e^{iq}}{k_{0}+k_{1}e^{iq}+k_{2}e^{-iq}} & \frac{2(k_{0}+k_{1}+k_{2})}{k_{0}+k_{1}e^{iq}+k_{2}e^{-iq}} \end{array}\right]$$ (2)

where *H*(*q*) is the effective Bloch Hamiltonian. We set the horizontal stiffness to be unit in the following (i.e., *k*_0_ = 1). The dimensionless eigenvalue of *H*(*q*) is λ(*q*) = *e*^−η(*q*)^ and corresponds to the decay factor of the static Rayleigh modes. The dispersion between η and *q* (or, equivalently, λ and *q*) is known as the decay spectrum, analogous to the dispersion relationship in wave dynamics (*35*, *42*). The spectrum of *H*(*q*) is real in reciprocal lattices (*k*_1_ = *k*_2_), owing to the quasi-Hermiticity character (see the Supplementary Materials), which implies that the applied load attenuates purely in the bulk material. In contrast, the decay factor λ is complex in nonreciprocal lattices (*k*_1_ ≠ *k*_2_) and carries an additional phase transition during spatial evolution (Fig. 1B). Note that we have a pair of eigenvalues, one with ∣λ_1_(*q*)∣ ≤ 1 and another with ∣λ_2_(*q*)∣ ≥ 1. They are denoted as the lower and upper branches and correspond to static Rayleigh modes applied on the left (*n* = 0) and right (*n* = ∞) edges, respectively. This subtle property is attributed to the symplectic nature of the Hamiltonian (*43*), since the system is invariant under the spatial inversion of *m*→−*m* and *n*→−*n*. The former is equivalent to *q*→−*q* and λ→λ^−1^, from which we have λ(−*q*) = λ^−1^(*q*). This spatial symmetry is analogous to the intrinsic particle-hole symmetry for mechanical systems (*44*), i.e., λ(−*q*) = −λ(*q*), with the minus operation replaced by the inversion operation. In addition, the system respects the TRS, i.e., Τ*H*(*q*)Τ^−1^ = *H*(−*q*) with Τ denoting the complex conjugation. With the combination of these symmetries, one can further deduce ∣λ_1_(*q*)λ_2_(*q*)∣ = 1 and arg[λ_1_(*q*)] = arg [λ_2_(*q*)], drawing a nontrivial implication relevant toward the non-Hermitian point-gap topology (*25*), as elucidated in the following.

### Non-Bloch bulk-edge correspondence in static mechanical metamaterials

**Fig. 2.**
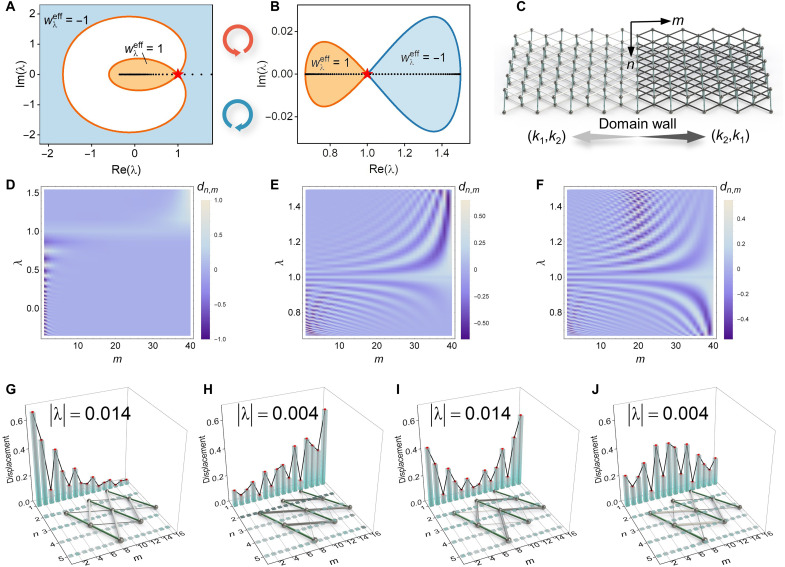
Non-Bloch BEC of the static mechanical metamaterials. (**A** and **B**) Decay spectra of the lattices under PBC (solid lines), SIBC (colored areas), and OBC (black dots), with the Bloch points labeled by red stars. The corresponding stiffnesses are (*k*_1_, *k*_2_) = (1,15) (A) and (0.01,0.03) (B), respectively. (**C**) Schematic of a domain wall connecting two metamaterials with (*k*_1_, *k*_2_) = (0.01,0.03) for *m* < 0 and (0.03,0.01) for *m* > 0. (**D** to **F**) Corresponding skin mode profiles of (A) to (C), where the *x* and *y* axes represent the nodal position along the *m* direction and the eigenvalue, respectively, with normalized displacement characterized by the colormap. (**G** to **J**) Zero modes for nonreciprocal single lattices (G and H) and domain walls (I and J), with stiffnesses (*k*_1_, *k*_2_) = (1,1.5) (G and I) and (2.1,1.7) (H and J), respectively.

The PBC spectra with different stiffness parameters are shown in Fig. 2 (A and B) (solid lines). The two branches form a loop geometry and degenerate at the exceptional point (*45*), λ = 1. For a reference point λ inside the closed loop, a point gap can be well defined. The corresponding point-gap topology is characterized by a spectral winding number (*25*), *w*_λ_ = (2π*i*)^−1^∮_BZ_*d*[ln det (*H* − λ)], where BZ denotes the Brillouin zone, *q* ∈ (−π, π]. Because λ and λ^−1^ form a reciprocal pair (see Materials and Methods), a spectrum with zero eigenvalue may induce singularity. Physically, the zero mode λ(*q*) = 0 implies that the static Rayleigh mode with wave number *q* is completely blocked on the boundary, giving rise to negligible perturbation inside the bulk. When *k*_1_ + *k*_2_ > 1, both the lower and upper branches encircle the origin and *w*_0_ = 2(−2) for *k*_1_ < (>)*k*_2_, whereas the two no longer encircle the origin and *w*_0_ = 0 whenever *k*_1_ + *k*_2_ is less than one (see the Supplementary Materials for details), indicating the topological phase transition. At the critical point *k*_1_ + *k*_2_ = 1, the lower branch λ_1_ crosses the origin and induces singularity of the upper branch (λ_2_→∞) due to the inversion symmetry. The corresponding wave number of the transition point is *q* = π, and its mechanical interpretation can be found in the Supplementary Materials. The phase transition of *w*_0_ draws substantial consequences. Because when *k*_1_ + *k*_2_ > 1, encircling of the origin for the lower branch exactly corresponds to the encircling of the infinity (λ→∞) for the upper branch on the extended complex plane $\bar{C}=C\cup \{\infty \}$. It means that for the upper branch, point gaps should lie outside the enclosed loop while prohibited inside the spectrum (such as the origin). In addition, the effective winding of the point gap outside the spectrum is opposite to that of the inner point defined above, i.e., $w_{\lambda }^{\mathrm{eff}}=w_{\lambda }$ or −*w_λ_* for *k*_1_ + *k*_2_ < 1 or *k*_1_ + *k*_2_ > 1, while its definition for the lower branch is unaltered.

Despite that the conventional bulk-edge correspondence (BEC) fails in the presence of non-Hermiticity, the emergent non-Bloch band theory restores the BEC both for the semi-infinite boundary condition (SIBC) and open boundary condition (OBC) (*25*, *26*). Colored areas in Fig. 2 (A and B) show the SIBC spectra for the two different phases (see the Supplementary Materials), i.e., one with *k*_1_ + *k*_2_ > 1 (Fig. 2A) and another with *k*_1_ + *k*_2_ < 1 (Fig. 2B). For either case, the SIBC spectrum is confined within the region with a nontrivial effective winding number and the corresponding eigenmodes are localized on either the bottom (*m*→0) or top (*m*→∞) boundary for $w_{\lambda }^{\mathrm{eff}}=1$ or −1, consistent with our analysis. For a metamaterial of finite size (i.e., *M* < ∞ and *M* is the system size in the vertical *m* direction), the OBC spectrum and eigenstates can be resolved by diagonalizing the real-space Hamiltonian (see the Supplementary Materials), as marked by solid dots in Fig. 2 (A and B). The OBC spectra are localized on the real axis with nontrivial topological phases ($w_{\lambda }^{\mathrm{eff}}\neq 0$). The corresponding eigenstate distributions are shown in Fig. 2 (D and E), where all bulk modes are localized on the boundary, a direct signature of the NHSE (*30*). To be more specific, the edge modes with eigenvalues less or larger than one are either bottom- or top-localized and form a bipolar NHSE (*46*), while the overall system is net-reciprocal owing to the inversion symmetry. This reciprocal skin effect is reminiscent of the ℤ_2_ skin effect protected by the anomalous TRS (*26*), because both systems carry paired skin modes with opposite localizations and the same decay length in real space and the reciprocally paired generalized Brillouin zones (GBZs) (*30*) in the momentum space (see the Supplementary Materials). The eigenstate of λ = 1 is fully delocalized and is called the Bloch point (*46*), as marked by the red stars in Fig. 2 (A and B). In fact, the point λ = 1 corresponds to the trivial rigid-body translation of the whole lattice along the *n* direction and is naturally delocalized into the bulk. We further investigate the effect of a domain wall that connects two metamaterials with switched stiffnesses, with (*k*_1_, *k*_2_) for *m* < 0 and (*k*_2_, *k*_1_) for *m* > 0, respectively (Fig. 2C). The SIBC spectrum of the entire system is identical to its constituent part (see the Supplementary Materials), and the skin modes under OBC are localized on the domain wall or two free boundaries (Fig. 2F). This is an interesting analog to the topological funnel effect of light transport (*47*).

**Fig. 3.**
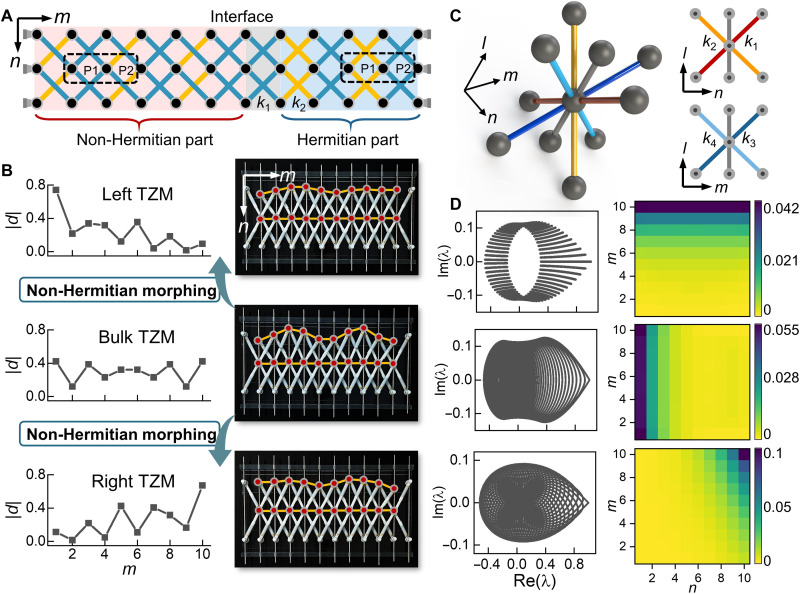
Non-Hermitian morphing of topological zero modes and higher-order skin effect in static Rayleigh model. (**A**) Schematic of the interface structure separating a mechanical analog of the non-Hermitian SSH model (shaded red area) and a Hermitian SSH model (shaded blue area). The dashed boxes mark the unit cells. (**B**) Spatial profiles of three topological zero modes (TZMs) are shown in the left panels, with the associated deformation plots shown in the right panels. The applied top-boundary loads are nearly blocked and are a direct evidence of the zero modes. The stiffnesses are (*k*_1_, *k*_2_) = (1,2), (1,1), and (2,1) from the top to bottom panels. The red circles mark the node position after deformation. (**C**) Schematic of the associate cell of the 3D layered square lattice with asymmetric diagonal couplings. The inset shows the projection of the associate cell on the *n*−*l* and *m*−*l* planes, respectively. (**D**) PBC spectra are shown in the left panels, and the corresponding mean amplitudes of all OBC bulk modes are shown in the right panels. The squeezing of bulk modes toward the boundaries or a corner signifies the higher-order skin effect. The stiffnesses are (*k*_1_, *k*_2_, *k*_3_, *k*_4_) = (1,1,2,4), (3,1,2,2), and (1,2,3,4) from the top to bottom panels.

It is known that zero modes enable extraordinary functionalities of metamaterials, such as the reduction of load unevenness and vibration isolation (*35*). Its combination with a skin mode can further enrich its applications. Figure 2 (G to J) depicts the zero mode profiles of single lattices and domain walls, with *N* × *M* = 5 × 16 and *N* being the system size in the *n* direction. Only the applied left zero modes with ∣λ∣→0 are shown in the figure, with their corresponding decay factors also listed. Note that it is almost impossible to obtain an exact zero-eigenvalue solution for a finite-sized system. With properly designed lattice configuration and stiffness parameters, loads with specific profiles can be effectively localized in these nonreciprocal metamaterials (see the Supplementary Materials for additional examples of the zero skin modes). Besides the above monoatomic lattice with only nearest-neighbor couplings, our model is general and is capable to study the multidimensional topological effects based on a purely passive modulation of the lattice geometry. For instance, enlarging the unit cell enables the realization of a mechanical analog of the (non-Hermitian) Su-Schrieffer-Heeger (SSH) model (Fig. 3A) that helps morph the spatial profile of the topological zero mode (Fig. 3B) (*48*), and extending our theory to 3D lattices (Fig. 3C) directly leads to a higher-order skin effect where all bulk modes are squeezed toward a boundary or even a corner (Fig. 3D) by tuning the bar stiffnesses (e.g., *k*_1_ ≠ *k*_2_ and *k*_3_ ≠ *k*_4_). The details of these two models and other examples, including a nonreciprocal lattice with long-range couplings (fig. S6) and a critical NHSE (*49*, *50*) in a bilayer lattice (fig. S9), are elucidated in the Supplementary Materials.

### Numerical and experimental validation

**Fig. 4.**
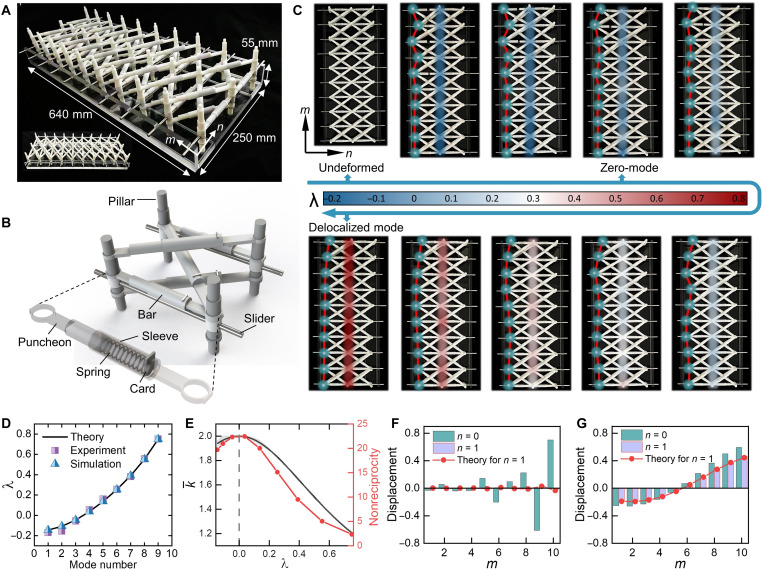
Experimental and numerical verifications of the NHSE. (**A**) Side view of the assembled truss–like lattice with size *N* × *M* = 3 × 10 and diagonal stiffnesses (*k*_1_, *k*_2_) = (1.36,0.68). Inset is the top view of the platform. (**B**) Schematic of a unit cell. (**C**) Experimental contour plots of the decay factors encoded in the colormap, with the bright dots and red lines denoting the prescribed boundary condition applied on the left boundary. (**D**) Theoretical, numerical, and experimental results of the nine decay factors. (**E**) Measured (gray filling) and predicted (solid line) relative interactions (the results are almost overlapped), with the red line referring to the nonreciprocity defined as *d*_0,10_/*d*_0,1_. (**F** and **G**) Displacement fields of the zero skin mode with the decay factor of −0.046 (F) and the delocalized bulk mode with the decay factor of 0.749 (G).

The above concept is then numerically and experimentally demonstrated in a truss-like lattice with *N* × *M* = 3 × 10 and (*k*_1_, *k*_2_) = (1.36,0.68), as shown in Fig. 4 (A and B) (see Materials and Methods and movie S2 for details of the implementation). The shaded columns in the 2nd to 10th panels in Fig. 4C qualitatively show the measured decay factors of nine eigen-modals with ∣λ∣ < 1 (the 10th modal is the trivial translation and is not shown here), with the undeformed configuration shown in the first panel. A color gamut unique to every eigen-modals indicates a collective deformation mode with a common decay factor for all lattice nodes, i.e., the static Rayleigh mode, while the squeezing of the applied load at *n* = 0 (which is an eigenstate of the OBC system) toward the top boundary signifies the skin effect. The averaged decay factors shown in Fig. 4D agree well with our theoretical prediction. The measured and analytically predicted relative interactions along opposite directions, defined as $\bar{k}=k_{m\to m+1}/k_{m+1\to m}=(\lambda^{-1}k_{1}+\lambda k_{2})/(\lambda^{-1}k_{2}+\lambda k_{1})$ following Eq. 1, are shown in Fig. 4E as the gray filling and black solid line, respectively. We find that $\bar{k}$ reaches its maximum near λ = 0 and is positively correlated to nonreciprocity that is characterized by the displacement ratio between *d*_0,10_ and *d*_0,1_, as marked by the red line in Fig. 4E. The measured displacement profiles of two typical modals with λ = −0.046 and 0.749 are shown in Fig. 4 (F and G) as the column bars, with the analytical prediction marked by the red dot-lines. The former is the zero mode, whose deformation is highly localized at *n* = 0, even if the applied load is unidirectionally concentrated because of the NHSE. The latter, which is near the Bloch point and is almost delocalized, induces a strong response inside the structure. Another branch of the skin modes with ∣λ∣ > 1 and an opposite spatial localization are readily obtained via space inversion. This is the first realization of (reciprocal) NHSE in a static system with purely passive control, to our best of knowledge.

**Fig. 5.**
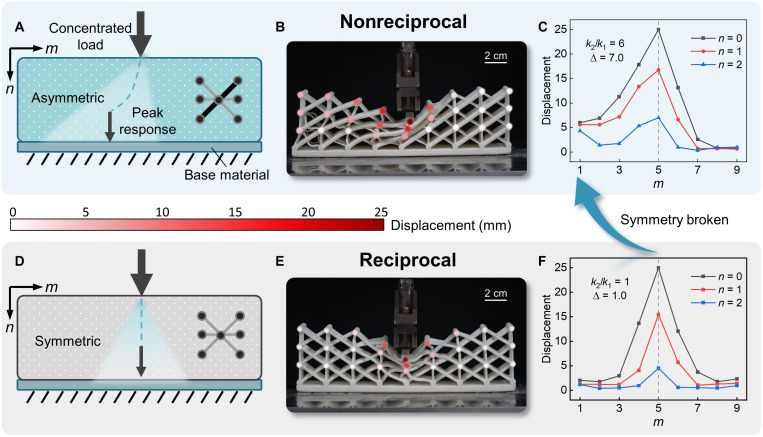
Dynamic signature of the NHSE in frame-like lattices. (**A** to **C**) Load transfer in a nonreciprocal lattice. (**D** to **F**) Load transfer in a reciprocal lattice. (A and D) Schematic of the transfer process of a concentrated load in the nonreciprocal (A) and reciprocal (D) lattices. (B and E) Deformation plots of the lattices under a concentrated displacement–controlled load applied on the top boundary, with the relative stiffnesses being *k*_2_/*k*_1_ = 6 (B) and 1 (E), respectively. (C and F) Corresponding nodal displacements of (B) and (E) and the parameter Δ characterize the nonreciprocity, while Δ = 1 corresponds to the reciprocal case (E).

For a trivial lattice with *k*_1_ = *k*_2_, the transfer process of a concentrated load applied on boundary is always symmetric owing to reciprocity (Fig. 5D), whereas it becomes unidirectionally localized for asymmetric lattice configuration (*k*_1_ ≠ *k*_2_), enabling high deformation shielding of a certain region (Fig. 5A). This counterintuitive phenomenon is actually a “dynamic” manifestation of the skin effect. Recall that a wave pulse in non-Hermitian dynamics is unidirectionally amplified toward a preferred direction while attenuated along the opposite during temporal evolution (*18*, *47*, *51*) which, in our static model, has been replaced by the spatial evolution along the *n* axis. This is demonstrated in the fabricated frame–like lattices with *N* × *M* = 4 × 9 (see Materials and Methods for details), where a quasi-static point load is applied on the center of the top boundary (*m* = 5 and *n* = 0) and the corresponding deformation fields are shown in Fig. 5 (B and E) with *k*_2_/*k*_1_ = 6 and *k*_2_/*k*_1_ = 1, respectively. It is clear that the deformation of the nonreciprocal case (Fig. 5C) is unidirectionally guided toward the left boundary, while it is almost symmetrically distributed in the reciprocal case (Fig. 5F and movie S3). The parameter Δ in Fig. 5 (C and F) quantifies the deflection strength of the bulk deformation and is defined as $\text{Δ}=\frac{1}{6\alpha }\sum_{n=0}^{2}\sum_{m=1}^{2}\rho (n,m)d_{n,m}/d_{n,10-m}$ (see Materials and Methods), which is enhanced with increasing stiffness contrast, *k*_2_/*k*_1_, owing to the nontrivial interaction between nonreciprocity and NHSE (*31*).

## DISCUSSION

Although non-Hermiticity itself is not limited to dynamic or active systems (*1*), its combination with nonreciprocity that catalyzes the skin effect is largely subjected to active actuations in previous studies (*7*, *8*, *16*, *18*, *33*, *47*, *52*), which inevitably injects/extracts additional energy into/from the closed systems. Going beyond wave dynamics, our model combines the non-Hermiticity, nonreciprocity, and band topology in an energy conserved system by purely passive modulation, upon which exotic topological phenomena have been be observed, such as the reciprocal skin effect and unidirectional localization. The presented model also provides an easily implemented platform for further exploration of novel non-Hermitian and nonreciprocal effects due to its strong robustness against perturbations and the flexible design capability.

## MATERIALS AND METHODS

### Lattice equilibrium equation in real and momentum spaces

For an inner node (*n*, *m*) with *n* > 0, its interaction with adjacent nodes is fully represented by the associate cell shown in Fig. 1A. The corresponding quasistatic equilibrium equation can be formulated as (note that only the horizontal nodal displacement is taken into account)

$$\begin{array}{rl} & k_{0}d_{n-1,m}+k_{0}d_{n+1,m}+k_{2}d_{n-1,m+1}+k_{2}d_{n+1,m-1}\\ & \;+k_{1}d_{n-1,m-1}+k_{1}d_{n+1,m+1}=k_{t}d_{n,m} \end{array}$$ (3)

Substituting the static Rayleigh deformation mode into the above equation, Eq. 1 can be readily obtained. Under the PBC along the *m* direction, Eq. 3 can be rewritten as a convolution form (*35*)

$$\sum_{n^{\prime }=n-1}^{n+1}\sum_{m^{\prime }=m-1}^{m+1}k_{n-n^{\prime },m-m^{\prime }}d_{n^{\prime },m^{\prime }}=\sum_{n^{\prime }=n-1}^{n+1}(k_{n-n^{\prime }}*d_{n^{\prime }})=0$$ (4)

where *k*_*n*−*n*′,*m*−*m*′_ denotes the linear interaction between adjacent nodes (*n*, *m*) and (*n*′, *m*′) and ∗ refers to the convolution operation between two periodic sequences, ***k*** and ***d***, with the size of *M* × 1 and *M* the periodicity of the crystal in the *m* direction. By virtue of the Fourier transform and the convolution theorem, we obtain the equilibrium equation in the momentum space (*30*)

$$\sum_{n^{\prime }=n-1}^{n+1}K_{n-n^{\prime }}(q)D_{n^{\prime }}(q)=0$$ (5)

where *q* = 2π*j*/*M* with integer *j* is the wave number, $K_{n-n^{\prime }}(q)=\frac{1}{\sqrt{M}}\sum_{m=0}^{M-1}k_{n-n^{\prime },m}e^{-iqm}$, and $D_{n^{\prime }}(q)=\frac{1}{\sqrt{M}}\sum_{m=0}^{M-1}d_{n^{\prime },m}e^{-iqm}=c\sqrt{M}e^{-\eta n^{\prime }}$ are Fourier coefficients. Then, Eq. 3 can be reformulated as an eigenvalue equation about the effective Bloch Hamiltonian *H*(*q*), that is, Eq. 2.

### Conserved/nonconserved quantity in static Rayleigh deformation model

In view of state evolution, a comparison between our Rayleigh attenuation–based closed static system and wave dynamic systems can be made. In conventional quantum dynamics, time evolution of an initial state ∣ψ(*t*_0_)〉 is governed by a state evolution operator $\hat{U}(t,t_{0})$ that is generated by the Hamiltonian $\hat{H}$, e.g., $\hat{U}(t,t_{0})=\exp[-i\hat{H}(t-t_{0})/\hbar ]$ in a stationary quantum system. When the Hamiltonian is Hermitian/non-Hermitian, the time evolution is unitary/nonunitary and preserves/changes the norm of the wave function, corresponding to a conservative/nonconservative probability. Such a characteristic also holds for classical waves, whereby the norm is usually related to their vibration energy (*53*).

In our static Rayleigh model, state evolution is taken along the spatial *n* dimension. Meanwhile, the temporal oscillation of a particle (*e*^−*i*ω*t*^) in wave dynamics is replaced by the spatial attenuation (*e*^−η*n*^) owing to the introduced imaginary time. Akin to the energy eigenstate of the Hamiltonian $\hat{H}$ where $\hat{U}(t,t_{0})$ is simply exp[−*iE*(*t*−*t*_0_)/ℏ], the state evolution operator for the Rayleigh eigen-solution ∣*d*(*n*)〉 is $\hat{U}(n,n_{0})=\exp[-\eta (n-n_{0})]$ (with *n*_0_ = 0 being assumed in the following analysis), by which the evolution of an initial state applied on the boundary (*n* = 0) is $|d(n)\rangle =\hat{U}(n,0)|d(0)\rangle$. In the coordinate representation along the *m* axis, we have 〈*m*∣*d*(*n*)〉 = *d*(*n*, *m*) = ∣*d*(*n*, *m*)∣*e*^*i*ϕ(*n*,*m*)^ = *e*^−η*n*^*d*(0, *m*) = *e*^−η*n*^∣*d*(0, *m*)∣*e*^*i*ϕ(0,*m*)^, where ϕ(*n*, *m*) is the phase factor of the state component at (*n*, *m*). In a Hermitian system with η ∈ ℝ, the former identity yields ∣*d*(*n*, *m*)∣ = *e*^−η*n*^∣*d*(0, *m*)∣ and ϕ(*n*, *m*) = ϕ(0, *m*), showing that although the norm ‖*d*(*n*, *m*)‖ is not conserved during spatial evolution, the phase factor ϕ(*n*, *m*) is still conservative. As is known, the decay factor is usually complex in a non-Hermitian system, and we have *d*(*n*, *m*) = ∣*d*(*n*, *m*)∣*e*^*i*ϕ(*n*,*m*)^ = *e*^−Re(η)*n*^∣*d*(0, *m*)∣*e*^*i*[ϕ(0,*m*)−Im(η)*n*]^, implying that the phase factor is no longer conserved during state evolution, i.e., ϕ(*n*, *m*) = ϕ(0, *m*) −Im (η)*n*.

It is thus clear that the conserved/nonconserved quantity in terms of Hermiticity/non-Hermiticity in our static Rayleigh model is the phase factor and not the norm in wave dynamics. Because the lack of the phase factor conservation has no effect on energy flow, even without energy exchange, non-Hermiticity can be invoked in our static system, through a proper design of the lattice configuration capable of obtaining additional phase transition for the Rayleigh mode.

The proposed time-space mapping is applicable to emulate a wide range of dynamic systems, ranging from de Broglie waves for electronics (*37*) to elastic waves in continuum media (*18*). As a result of the abandoned energy gain/loss effect and external manipulation, our model is particularly suitable for the design of complex experiments that are difficult to implement in a dynamic system, such as a NHSE experiment. Furthermore, the static nature of the concerned system provides additional robustness against damping and dissipation compared with a wave dynamic system when conducting an experimental measurement (*54*–*56*).

### Inversion symmetry–protected reciprocal skin effect

Note that the eigenvalues of *H*(*q*) are always in reciprocal pairs (i.e., λ and λ^−1^), owing to the 2D inversion symmetry (or the overall reciprocity) of the lattice structure. As a result, for a static Rayleigh mode applied on the left edge (*n* = 0) with a decay factor λ_L→R_ = *d*_*n*+1,*m*_/*d*_*n*,*m*_, there must be a reciprocal counterpart applied on the right boundary (*n* = ∞) with $\lambda_{R\to L}=d_{n,m}/d_{n+1,m}=\lambda_{L\to R}^{-1}$, forming a reciprocal pair embedded in *H*(*q*) (fig. S1). To clarify this, we define a compound Hamiltonian $\bar{H}=H+H^{-1}$, with its eigenvalue $\bar{\lambda }=\lambda +\lambda^{-1}$ containing the reciprocal pair simultaneously and the same eigenvector for its subpart. For symmetric configuration with *k*_1_ = *k*_2_ = *k*, we always have a reducible form of $\bar{H}(q)=(2+4k)/[1+2k\cos(q)]I$, where *I* is the 2 × 2 identity matrix. Thus, the system is decoupled with a twofold degenerate real spectrum. It is noted that the degeneracy can be lifted with the broken of symmetry (i.e., *k*_1_ ≠ *k*_2_) owing to the coupling of the two subparts. In either case, the inversion symmetry is maintained for the whole system, i.e., $P\bar{H}(q)P^{-1}=\bar{H}(-q)$ where *P* = σ*_x_* is the Pauli matrix. Hence, the energy levels at the opposite momenta are degenerate and form a parity pair, and the two branches coalesce at the inversion-invariant momenta in the BZ, i.e., Γ*_a_* = 0 and Γ*_b_* = π (see fig. S2). As can be demonstrated by the non-Bloch band theory, the Hamiltonian with inversion symmetry is trivialized, and the skin effect is restrained because the spectral winding number is always zero (*57*). However, we emphasize that this is not the case for our model where the Hamiltonian is protected by additional PT symmetry, $(\mathrm{PT})\bar{H}{\left(\mathrm{PT}\right)}^{-1}=\bar{H}$, and the system lies in the PT-broken phase as long as *q* ≠ 0 and λ ≠ 1. Consequently, the eigenvalues form complex conjugate pairs with two branches coinciding with each other and inheriting opposite windings with respect to a point gap. As a result, the global winding number is zero. Furthermore, the eigenvalues of the whole system under the OBC are either confined within or without the PBC spectrum for distinct topological phases (fig. S2), consistent with the non-Bloch band theory and our redefined point gap. Crucially, each OBC eigenvalue $\bar{\lambda }$ is twofold degenerate and consists of a parity pair: One is a bottom-localized skin mode that attenuates along the +*n* axis, while the other is top-localized and attenuates along the −*n* axis, ensuring overall reciprocity of the entire system.

The reciprocal skin effect in our closed static system is phenomenologically similar to the ℤ_2_ skin effect with Kramers degeneracy (*26*) in an open dynamic system because in both systems, oppositely localized skin modes emerge in pair and the systems are net-reciprocal. The minor difference between them lies in their corresponding protected symmetries: The reciprocal skin effect in our model and the ℤ_2_ skin effect are protected by the space inversion symmetry and the anomalous (internal) TRS (*58*), respectively. The similarity between the ℤ_2_ skin effect and our model can also be seen from the geometry of GBZ in the momentum space, as outlined in the Supplementary Materials.

### Numerical simulations

In contrast with wave dynamics whose eigen-modal can be excited by pulsed excitation with a certain frequency, the capability for exciting an eigenstate with a common decay factor for all lattice nodes in our static system is closely related to the prescribed load applied on the boundary. This is reasonable because the spacetime transform converts the initial value problem to a boundary value problem. Here, we conduct numerical simulations using the analogous Rayleigh-Ritz method in structural dynamics (*42*). The displacement load applied on the left boundary (*n* = 0) is an eigen-modal **ψ** with an associated decay factor η, while the load applied on the right boundary (*n* = 2) is e^−2^^η^**ψ**. Then, the collective deformation mode can be excited for the middle column, *n* = 1, and the decay factor can be determined unambiguously by measuring the relative displacement between adjacent columns, e.g., *d*_1,*m*_/*d*_0,*m*_. We simulate the lattice model shown in Fig. 1A through a truss-like lattice with size *N* × *M* = 3 × 10 in the commercial finite-element-method software Ansys. All nodal degrees of freedom (DoFs) except the horizontal translation are restricted, and the prescribed displacement fields obtained analytically above are applied on both the left and right boundaries. The measured decay factor shown in Fig. 4D is obtained from the average of 10 nodes, i.e., $\lambda =\frac{1}{M}\sum_{i=1}^{M}d_{1,i}/d_{0,i}$. The traction-free boundary condition is adopted for the top and bottom boundaries (i.e., *m* = 1 and *m* = *M*). Because the truss is a stretch-dominant structure with its deformation governed by axial tension and compression (*59*), the linear stiffness of the bar can be formulated as *k* = *EA*cos^3^(θ) under the infinitesimal deformation, where *EA* and θ denote the tensile section modulus and the inclined angle between the bar and horizontal direction, respectively. For horizontal (θ = 0°) and diagonal (θ = 45°) bars, the stiffnesses are *k*_h_ = (*EA*)_h_ and $k_{d}=\sqrt{2}{\left(EA\right)}_{d}/4$, respectively, and the relative stiffnesses between the two are *k_i_* = 0.35(*EA*)_d*i*_/(*EA*)_h_, with *i* = 1,2. Therefore, the stiffness parameters can be adjusted flexibly by modulating the cross-sectional area of bars, which is convenient for practical implementation.

### Experimental measurements

The experimental platform shown in Fig. 4 (A and B) is a stretch-dominant truss-like lattice with size *N* × *M* = 3 × 10, where the nodes are replaced by cylindrical pillars and the bars between adjacent pillars are connected by linear springs with sleeves outside the springs to prevent buckling. One end of sleeve is slotted, and a small card is inserted to fix the spring, while the other end of the spring is glued to the puncheon. To prevent the crossing of the diagonal bars in a unit cell while maintaining the balance of the pillars, a pair of bars connecting two pillars is designed along a diagonal direction (e.g., along *k*_1_), while a single bar in another diagonal direction connects the middle points of the pillars (Fig. 4B). Pillars in the same row (i.e., with the same *m*) are connected by horizontal sliders with both ends penetrating through the wings of the U-shaped platform, from which extra DoFs are restricted and the pillars can only translate horizontally. On the left and right boundaries of the structure, the prescribed displacement fields are applied through the match between the bottom end of the cylindrical pillars and the holes drilled on the baseboard of the U-shaped platform (movie S2). Akin to the numerical simulations, the decay factor is obtained by taking the average of 10 measured data. The relative stiffnesses between the diagonal and horizontal bars are *k_i_* = cos^2^(θ)*k*_d*i*_/*k*_h_ with *i* = 1,2, where *k*_d*i*_ and *k*_h_ represent the diagonal and horizontal spring constants, respectively. In this experiment, we use nominally the same springs for all directions. Hence, the relative stiffnesses are *k*_1_ = 2 × cos^2^(θ) = 1.36 and *k*_2_ = cos^2^(θ) = 0.68 with θ = 34.5° (note that a pair of parallel bars are placed along *k*_1_).

In the second experiment, we use the 3D printing technique to manufacture the frame-like lattice, and set the diagonal beams along two directions (i.e., *k*_1_ and *k*_2_) with different numbers and sectional areas to tailor the nonreciprocity. The relative stiffness is defined as *k*_2_/*k*_1_ = *n*_2_*r*_2_/*n*_1_*r*_1_, where *n_i_* and *r_i_* respectively denote the number of beams and sectional diameters along the two diagonal directions. Besides the models shown in Fig. 5, we also construct another two frame lattices with distinct reciprocity, as shown in fig. S3. The experiment aims at a qualitative demonstration of the asymmetric deformation distribution and subsequent deformation shielding capability in the nonreciprocal lattices with *k*_1_ ≠ *k*_2_. Note that although the lattice node is restricted to only translate along the *n* direction in theory, we release the restriction in this experiment for easier implementation. To justify the release of the restriction of nodal DoF, we simulate the frame-like lattices of nominally the same geometry, stiffness parameters, and loading conditions as the fabricated samples, one with single DoF and another with double DoF (i.e., the restriction released). The results shown in fig. S3 indicate that the bulk static deformation is always symmetric for the reciprocal lattices with *k*_1_ = *k*_2_, independent of the restriction of DoF, whereas the bulk deformation skews toward the boundary and enables the deformation shielding at the opposite boundary whenever *k*_1_ ≠ *k*_2_. The measured deflection strength Δ is found to increase monotonously with the increment of the stiffness contrast, *k*_2_/*k*_1_, for both the single and double DoF lattices, and provides added support of asymmetric deformation distribution and subsequent deformation shielding. The bottom of the frame is fully anchored on the base plate of the tensile testing machine, and the collet of the testing machine clamps the card fixed on the top of the beam to apply the compressive load. The nodal displacements are measured by the digital image correlation method. The defined deflection strength Δ only accounts for the nodal deformation of the left two boundary columns and the right two boundary columns to reduce the edge effect adjacent to the loading point, where ρ(*n*, *m*) is the weighting factor for different nodes and α is the normalization coefficient for Δ of the reciprocal lattice (Fig. 5E). The results listed in this article are obtained for ρ = 1 and α = 0.976.

## Supplementary Materials

### This PDF file includes:

Supplementary Text Figs. S1 to S9 Legends for movies S1 to S3 References
