# Supplementary material for: Non-Hermitian topology in static mechanical metamaterials
Source: Sci Adv. 2023 Jul 5;9(27):eadf7299. doi: 10.1126/sciadv.adf7299 (PMC10321739; doi:10.1126/sciadv.adf7299)
Supplement: Supplementary file 1 — Supplementary Text Figs. S1 to S9 Legends for movies S1 to S3 References [file sciadv.adf7299_sm.pdf]

Science Advances
AAAS

Supplementary Materials for

Non-Hermitian topology in static mechanical metamaterials

Aoxi Wang *et al.*

Corresponding author: Chang Qing Chen, chencq@tsinghua.edu.cn

Sci. Adv. **9**, eadf7299 (2023)
DOI: 10.1126/sciadv.adf7299

The PDF file includes:

Supplementary Text
Figs. S1 to S9
Legends for movies S1 to S3
References

Other Supplementary Material for this manuscript includes the following:

Movies S1 to S3

Supplementary Text

1. Quasi-Hermiticity for reciprocal lattices

As mentioned in the main text, the effective Hamiltonian of the mechanical metamaterials shown in Fig. 1A can be expressed as

$$H(q)=\begin{bmatrix} 0 & 1 \\ -\dfrac{k_0+k_1e^{-iq}+k_2e^{iq}}{k_0+k_1e^{iq}+k_2e^{-iq}} & -\dfrac{2(k_0+k_1+k_2)}{k_0+k_1e^{iq}+k_2e^{-iq}} \end{bmatrix} \tag{S1}$$

where the non-reciprocity depends on the relative stiffnesses between the two diagonal bars, i.e., k_1/k_2. At the reciprocal limit, i.e., $k_1=k_2=k$, the above formula can be simplified as

$$H_{\text{rec}}(q)=\begin{bmatrix} 0 & 1 \\ -1 & \dfrac{2+4k}{1+2k\cos(q)} \end{bmatrix} \tag{S2}$$

where we have set $k_0=1$ for the sake of simplification. One can check that the spectrum of the above Hamiltonian is real as guaranteed by its quasi-Hermiticity. A matrix or operator H is defined as quasi-Hermiticity only if there is a Hermitian and positive definite matrix or operator, Γ, such that one has $\Gamma H=H^{\dagger}\Gamma$ (*1*). It can be proved that the spectrum of a quasi-Hermitian Hamiltonian is entirely real, a rigorous proof can be found in Ref (*60*).

To illustrate this viewpoint, we first specify $\chi=2+4k$ and $A(q)=1+2k\cos(q)$, and the inequation $\chi^2\geq(2A)^2$ is always true owing to the fact that k is real, from which the transform matrix can be defined as

$$\Gamma=\begin{bmatrix} \chi & -2A \\ -2A & \chi \end{bmatrix} \tag{S3}$$

Γ is Hermitian since both χ and A are real. The spectrum of Γ is $\gamma_{1,2}=\chi\pm 2|A(q)|\geq 0$, and the zero point corresponds to the trivial case, $q=0$, where a pair of real eigenvalues with $\lambda_1=\lambda_2=1$ can be obtained. Therefore, Γ is both Hermitian and positive definite (excluding $q=0$), and one can further check that

$$\Gamma H_{\text{rec}}=\begin{bmatrix} 2A & -\chi \\ -\chi & \dfrac{\chi^2-2A^2}{A} \end{bmatrix}=H_{\text{rec}}^{\dagger}\Gamma \tag{S4}$$

which confirms the quasi-Hermiticity and the fact that the entire spectrum of $H_{\text{rec}}(q)$ is real. Intuitively, a static Rayleigh mode propagating in a lattice material with symmetric configuration (i.e., $k_1=k_2$) cannot accumulate additional phase factors and λ is real. Whereas it is invalid for asymmetric lattices with $k_1\neq k_2$, since the Hamiltonian is no longer quasi-Hermitian and may have a complex spectrum, as clarified in the main text.

2. Winding number for zero-eigenvalue

The spectral winding number pertaining to the zero-eigenvalue $\lambda=0$ can be simplified as

$$w_0=\frac{1}{2\pi i}\oint_{|z|=1}\Lambda-\overline{\Lambda}dz \tag{S5}$$

where we have set $z=e^{iq}$ and the complex functions Λ and $\overline{\Lambda}$ are defined as

$$\Lambda(z)=\frac{k_2z^2-k_1}{z\left(k_2z^2+z+k_1\right)} \qquad \overline{\Lambda}(z)=\frac{k_1z^2-k_2}{z\left(k_1z^2+z+k_2\right)} \tag{S6}$$

Eq. (S5) can be solved analytically with the residue theorem. It is straightforward to see that $z_1=\overline{z}_1=0$ are the respective single pole of Λ and $\overline{\Lambda}$. The corresponding residues are $\mathrm{Res}(\Lambda,z_1)=\mathrm{Res}(\overline{\Lambda},\overline{z}_1)=-1$, meaning that the two cancel each other out and have no effect on the integral. The remaining two poles of Λ and $\overline{\Lambda}$ are

$$z_{2,3}=\frac{-1\pm\sqrt{1-4k_1k_2}}{2k_2} \qquad \overline{z}_{2,3}=\frac{-1\pm\sqrt{1-4k_1k_2}}{2k_1} \tag{S7}$$

We divide the stiffness parameters into two categories: (i) $4k_1k_2>1$ and (ii) $4k_1k_2<1$. In the former case (i), $z_{2,3}$ and $\overline{z}_{2,3}$ form a complex conjugate pair, and we have $\left|z_2^2\right|=\left|z_3^2\right|=z_2z_3$ and $\left|\overline{z}_2^2\right|=\left|\overline{z}_3^2\right|=\overline{z}_2\overline{z}_3$ according to Vieta theorem. Moreover, $\left|z_{2,3}\right|^2<1$ and $\left|\overline{z}_{2,3}\right|^2>1$ are simultaneously satisfied when $k_1<k_2$, which indicates that only $z_{2,3}$ are located within the integration path $|z|=1$. As a result, Eq. (S5) can be simplified as $w_0=\mathrm{Res}(\Lambda,z_2)+\mathrm{Res}(\Lambda,z_3)$ where $\mathrm{Res}(\Lambda,z_{2,3})=\left(k_2z_{2,3}^2-k_1\right)/\left[k_2z_{2,3}\left(z_{2,3}-z_{3,2}\right)\right]$. Hence, we obtain a closed-form expression of w_0 as $w_0=1+k_1/(k_2z_2z_3)=2$. Similarly, we can obtain $w_0=-2$ for $k_1>k_2$. In the latter case (ii), all four roots $z_{2,3}$ and $\overline{z}_{2,3}$ are negative real numbers with $|z_2|<|z_3|$ and $|\overline{z}_2|<|\overline{z}_3|$. We first prove that only two of the four roots lie inside the unit circle, $|z|=1$. Note that it is not possible that all four roots lie within the unit circle, otherwise a contradiction arises, i.e., $z_2z_3<1$ with $k_1<k_2$ and $\overline{z}_2\overline{z}_3<1$ with $k_1>k_2$. Moreover, it is also impossible for three of the four roots to have modulus less than 1. To gain this point of view, assume that if $|z_{2,3}|<1$, $|\overline{z}_2|<1$ and $|\overline{z}_3|>1$, then we have $z_3>-1$ and $\overline{z}_2>-1$. The former condition implies that (a) $k_1+k_2>1$ and $2k_2>1$, while from the latter condition we have (b1) $k_1+k_2<1$ and $2k_1<1$ or (b2) $2k_1>1$. While condition (b1) contradicts (a) and should be discarded, the combination of (b2) and (a) directly induces $4k_1k_2>1$, which is inconsistent with the major premise. From a similar procedure, we can prove that it is impossible for the other three roots z_2 and $\overline{z}_{2,3}$ to lie within the unit circle as well. Furthermore, suppose that if only a single root (e.g., z_2) lies within the unit circle, which is equivalent to $z_2>-1$, $z_3<-1$ and $\overline{z}_2<-1$. From $z_2>-1$ we have (c1) $k_1+k_2<1$ and $2k_2<1$ or

(c2) $2k_2 > 1$, while the condition of $z_3 < -1$ and $\bar{z}_2 < -1$ indicates that (d1) $k_1 + k_2 < 1$ and $2k_2 > 1$ or (d2) $2k_2 < 1$ and (e) $k_1 + k_2 > 1$ and $2k_1 < 1$, respectively. One can verify that the above three sets of conditions (c–e) cannot be satisfied simultaneously. Finally, suppose if all four roots are located outside of the unit circle, which is equivalent to $z_2 < -1$ and $\bar{z}_2 < -1$. The former condition requires that $k_1 + k_2 > 1$ and $2k_2 < 1$, while the latter indicates that $k_1 + k_2 > 1$ and $2k_1 < 1$, which contradicts each other. Thus, the assumption cannot hold.

To sum up, as long as $4k_1k_2 < 1$, there are two and only two of the four roots $z_{2,3}$ and $\bar{z}_{2,3}$ are located within the unit circle, which can only be z_2z_3, $\bar{z}_2\bar{z}_3$ or $z_2\bar{z}_2$. When the two roots are z_2z_3, we have $z_3 > -1$ and $\bar{z}_2 < -1$, or equivalently, $k_1 + k_2 > 1$ and $k_1 < k_2$ (e.g., $k_1 = 0.25$ and $k_2 = 0.77$). Whereas we have $\bar{z}_3 > -1$ and $z_2 < -1$ when the two roots are $\bar{z}_2\bar{z}_3$, which indicates that $k_1 + k_2 > 1$ and $k_1 > k_2$ (e.g., $k_1 = 0.77$ and $k_2 = 0.25$). Finally, when the two roots are $z_2\bar{z}_2$, we have $\bar{z}_2 > -1$ and $z_2 > -1$, which simply requires that $k_1 + k_2 < 1$ (e.g., $k_1 = 0.6$ and $k_2 = 0.2$). For the first two cases (z_2z_3 or $\bar{z}_2\bar{z}_3$), one can get that $w_0 = 2$ or $w_0 = -2$, while we find $w_0 = 0$ for the third case ($z_2\bar{z}_2$) by substituting Eq. (S7) to the Residue Theorem.

With all the cases analyzed above, we obtain the phase diagram elucidated in the main text.

3. Mechanical zero mode and force-amplitude relationship

One of the most important features of the static Rayleigh model is zero mode, i.e., $\lambda = 0$, which shows that boundary loads can be fully blocked from penetrating the bulk. Zero modes can appear in highly non-local lattices, e.g., the static non-reciprocal metamaterial shown in Fig. 1A. With zero modes, a metamaterial can have special functions like vibration isolation and flattening of loading profile.

To demonstrate the capability of the zero mode in reduction of loading unevenness, we consider three planar lattices of the size $N \times M = 10 \times 20$, i.e., a square lattice without a zero mode, a short-range lattice with one zero mode, and a long-range lattice with two zero modes, as shown in Fig. S4A for the lattice configurations and stiffness parameters. In each lattice, the horizontal stiffnesses are unitized and every vertical or diagonal stiffnesses are the same (i.e., the lattices are symmetric). Their corresponding decay spectra are shown in Fig. S4B, where the real spectra are due to the quasi-Hermiticity of their Hamiltonians (see text 1). Three loading patterns (i.e., zig-zag, Gaussian, and random form) of the same average value $\bar{d}$ are applied on the lattice boundary $n = 0$, and the bulk displacement responses at $n \geq 1$ are extracted to calculate the roughness Δ of the deformation, which is defined as $\Delta_n = \left\| d_{n,m} - \bar{d} \right\|_2$ and $\left\| \cdot \right\|_2$ is the 2-norm. Figure S4D depicts the extracted nodal displacements at $n = 0$, 1 and 2. It is found that the lattices with zero mode have a much better capability of reducing load unevenness. In particular, the zig-zag loading is almost completely flattened after only passing through two layers from $n = 0$ to 2 within the lattices with one or two zero modes (see, the first column of Fig. S4D), while the unevenness of deformation profile is still evident in the square lattice. The calculated Δ further demonstrates the flattening behavior of the zero modes, as shown in Fig. S4C. With such load flattening capacity, a lattice metamaterial with zero modes can be designed as a mechanical filter to eliminate the noise of a loading signal, or alleviate stress concentration, such as the flattening of the Gaussian packet (Fig. S4C and D).

Moreover, richer zero modes can be achieved by introducing non-reciprocity into the lattices, e.g., $k_1 \neq k_2$ in the short-range lattice. The reason lies in that the eigenstate of a zero mode is no longer restricted to an extended Bloch wave and owing to the non-Hermitian skin effect (NHSE), can be localized on boundary. Hence, non-reciprocal lattices are advantageous over reciprocal lattices in blocking an acentric load away from the center of a lattice, especially for a concentrated load applied on a single node. To illustrate this point, we take the short-range lattice as an example, with three different sets of diagonal stiffnesses of (i) $(k_1, k_2) = (1,5)$, (ii) $(5,1)$, and (iii) $(3,3)$. The first two are non-reciprocal lattices with (i) bottom- or (ii) top-localized skin modes, while the third (iii) is reciprocal and is shown for comparison. A point load is applied on the node $(n,m) = (0,1)$ or $(0,20)$, i.e., the bottom or top boundary, and the node at $(n,m) = (0,2)$ or $(0,19)$ is preloaded to enhance localization of the point load. The right boundary ($n = 9$) is anchored to prevent rigid-body translation.

Figure S5A shows the deformation profiles at $n = 1$ for the three lattices, where a maximum at $m = 2$ or $m = 19$ can be observed owing to the strong diagonal interactions of the short-range lattices. The load applied on the bottom boundary decays significantly upon reaching $n = 1$ in (i) (dark red line), thanks to its bottom localized zero mode, while a strong response is observed in (ii) because the bottom load is not the eigenstate of its zero mode (light red line). By contrast, the top load resembles the zero eigenmode of (ii) and decays rapidly (dark blue line), while it is not an eigenstate of (i) and a strong response at $n = 1$ is observed (light blue line). The inset of Fig. S5A shows the decay profiles along the *n* axis of the bottom load at $m = 1$ and top load at $m = 19$, and again, rapid attenuation emerges for the bottom load in (i) and the top load in (ii) (marked by the squares), indicating effective localization of point load by the zero skin mode. The blocking of other loading patterns in zero mode can be achieved with an elaboration of the lattice configuration. For example, a domain wall separating (ii) (bottom placed) and (i) (top placed) can be used to block a point load applied even away from boundary, as dictated by its skin mode profile shown in Fig. 2J.

Furthermore, in terms of the non-Bloch band theory, the zero skin mode of a finite lattice can be predicted from the bulk-edge correspondence (BEC). For example, only a non-reciprocal lattice with its PBC spectral loop encircling the origin can harbor a zero mode under the OBC, which is pumped to boundary by the NHSE. A criterion to determine zero mode (or any eigenvalue to be considered) for an OBC system is the generalized Brillouin zone (GBZ) method (see details of GBZ in text 7). The characteristic equation of the short-range lattice is $f(z,\lambda) = (k_1 z^2 + z + k_2)\lambda^2 - 2z(1 + k_1 + k_2)\lambda + k_2 z^2 + z + k_1 = 0$ with $z = e^{iq}$ the complex wavenumber, which yields two solutions $z_1(\lambda)$ and $z_2(\lambda)$ for a given decay factor λ. Under the condition of $|z_1(0)| = |z_2(0)|$, a zero mode at the thermodynamic limit ($M \to \infty$) is guaranteed, from which we obtain the critical stiffness of the zero mode, i.e., $4k_1 k_2 > 1$. This criterion is consistent with the spectral winding number obtained in text 2, because the PBC spectrum winds around the origin when $k_1 + k_2 > 1$ and we have $1 \leq 2\sqrt{k_1 k_2} \leq k_1 + k_2$. The equality holds at the reciprocal limit with $k_1 = k_2 = 1/2$, for which the Bloch BEC is restored and the zero mode is delocalized into the bulk. Moreover, the skin mode $d_{0,m} \sim z^m$ has an inverse decay length (IDL)

$\gamma = -\ln(|z|)$, with $\gamma = \frac{1}{2}\ln(k_2/k_1)$ for the zero mode. We can then tune the skewing position and the localization strength of the zero mode by varying the relative stiffness k_2/k_1. Figure S5B shows the dispersion of the IDL with increasing k_2/k_1 (black line, and only the bottom localized zero modes with $k_2/k_1 > 1$ are shown for the sake of brevity), while the numerically measured decay rate (red line) is obtained from the inverse participation ratio (IPR) of the OBC eigenstate with a sufficiently small decay factor ($\lambda \to 0$). The IPR is defined as (*61*)

$$I_\alpha = \frac{\sum_m |d_m|^4}{\left(\sum_m |d_m|^2\right)^2} \tag{S8}$$

The IPR approaches zero for an extended Bloch wave, while a fully localized edge state has an IPR nearing the upper bound 1 (dashed gray line). The bottom localization of the zero skin mode is enhanced with increasing k_2/k_1, as further confirmed by the normalized displacement contours shown in Fig. S5C. Note that another branch of the top localized zero modes, which is applied on the right boundary, can be blocked as well, thanks to the inversion symmetry. Moreover, a local defect can be constructed to trap zero mode (*47*), such as a domain wall geometry shown in the main text. This again shows that, with non-Bloch band theory, our static metamaterial system provides an avenue for the design of desired mechanical functionalities from a topological perspective.

Force-amplitude relationship (FAR) is an effective method to qualitatively analyze the propagation of bulk deformation in the static Rayleigh model. We take the lattice material shown in Fig. 1A as an example to demonstrate this idea. In fact, FAR is a general method applicable to even more complicated lattice structures. When an external load is applied on the left boundary of the lattice, the exerted force $F_{n,m}$ of an inner point (n,m) with $n > 1$ can be derived merely from its left three neighbors, $(n-1,m)$, $(n-1,m-1)$, and $(n-1,m+1)$, given by

$$F_{n,m} = \lambda^{n-1}\left(k_1 e^{-iq} + k_2 e^{iq} + 1\right)e^{iqm} \tag{S9}$$

where the static Rayleigh solution is adopted and the amplitude is unitized for simplification. For the reciprocal lattices with $k_1 = k_2 = k$, Eq. (S9) can be simplified as

$$F_{n,m} = \lambda^{n-1}\left[1 + 2k\cos(q)\right]e^{iqm} = \left[1 + 2k\cos(q)\right]d_{n-1,m} \tag{S10}$$

The above formulation implies that $F_{n,m}$ is in phase with the Bloch mode, with a common phase factor $\varphi_{n,m} = qm$. It can be shown that the amplitude $A(q) = 1 + 2k\cos(q)$ of Eq. (S10) gives rise to the propagation law of the static Rayleigh modes in a qualitative manner, which can be summarized as follows:

(i) When $|A(q)|$ decreases with increasing wavenumber q, i.e., $d|A(q)|/dq < 0$, the attenuation rate of the static Rayleigh mode is enhanced with the increment of q.

(ii) When $\left|A(q)\right|$ increases with q, the attenuation rate of static Rayleigh mode with short wavelength decreases, which is a manifestation of the reverse Saint-Venant (RSV) edge effect (*35*).

(iii) Negative $A(q)$ implies that the direction of driving forces $F_{n,m}$ exerted on point (n,m) is opposite to $d_{n-1,m}$, which can be regarded as an indicator of the displacement modal inversion, i.e., the phase difference of Bloch modes between two adjacent columns n and $n-1$ is $\varphi_{n,m}-\varphi_{n-1,m}=\pi$.

(iv) In particular, $A(q)=0$ is equivalent to $\lambda(q)=0$, which is a direct indication of a zero mode and the static Rayleigh mode with wavenumber q is completely blocked at the boundary.

In general, above conclusions are not fully applicable to non-reciprocal lattices owing to an additional phase transition of the static Rayleigh modes. Nevertheless, a zero mode ensures that the deformation is completely blocked at the boundary, thus the fourth aspect (iv) remains validate even for the non-reciprocal systems with $k_1 \neq k_2$. From Eq. (S9), the force amplitude exerted on $n=1$ is $A(q)=k_1e^{-iq}+k_2e^{iq}+1$, which can be further expanded as $A(q)=1+(k_1+k_2)\cos(q)+(k_2-k_1)\sin(q)i$ by Euler's formula. Now we can see that the zero mode $A(q)=0$ specifies that $q=\pi$ and $k_1+k_2=1$, consistent with the analytical result in the main text.

4. Non-reciprocal lattices with long-range interactions

To further exploit the underlying non-Hermitian mechanisms behind these metamaterials, we design a class of long-range lattices with the next-nearest-neighbor interactions, and their associate cell is pictorially shown in Fig. S6A. The configuration can be realized in practice by subtly adjusting the height of lattice nodes along the *l*-axis shown in Fig. 1A, e.g., increasing the height of a portion of red nodes for a certain distance while keeping the other nodes unchanged. These static lattice materials form an analogy to the extended HN model with farther hopping amplitudes (*27*) as well as the mass-spring model with long-range interactions (*62*). Stiffnesses of diagonal bars are k_1, k_2, k_3 and k_4 respectively, while the horizontal stiffness k_0 is set to the unit value (Fig. S6A). The effective non-reciprocity is presented when either $k_1 \neq k_2$ or $k_3 \neq k_4$. Akin to Eq. (S1), the Bloch Hamiltonian of the long-range lattices under PBC can be written as

$$H(q)=\begin{bmatrix} 0 & 1 \\ -\dfrac{1+k_1e^{-iq}+k_2e^{iq}+k_3e^{-2iq}+k_4e^{2iq}}{1+k_1e^{iq}+k_2e^{-iq}+k_3e^{2iq}+k_4e^{-2iq}} & \dfrac{2(1+k_1+k_2+k_3+k_4)}{1+k_1e^{iq}+k_2e^{-iq}+k_3e^{2iq}+k_4e^{-2iq}} \end{bmatrix} \quad \text{(S11)}$$

The inherent high non-locality of these metamaterials endows them with abundant non-Hermitian topological characteristics. Figure S6B to G shows the decay spectra under different boundary conditions and the skin mode profiles, respectively. Note that only the eigenvalues with modulus less than 1 (corresponding to the static Rayleigh mode applied on the left boundary) are shown for brevity. Compared with the short-range lattices discussed in the main text, the spectrum of the long-range lattices shows intricate winding numbers, e.g., $w_\lambda=2$ in

Fig. S6B and $w_\lambda = \pm 1$ in Fig. S6C (the effective winding number w_λ^{eff} is coincident with w_λ since only the lower spectra are presented). With k_1 increasing, the winding orientation of the PBC spectrum reverses from counterclockwise to clockwise, implying the migration of the OBC skin modes from the bottom to top boundaries.

In texts 5 and 6, we analytically calculate the spectrum and eigenstates of the long-range lattices under SIBC and OBC, and the results are fully consistent with the non-Bloch BEC. Especially, for Fig. S6B, bottom localized SIBC edge states with two-fold degeneracy are observed within the domain of $w_\lambda = 2$ (dark orange area), while bidirectional SIBC edge states arise in Fig. S6C (light orange and cyan areas). Meanwhile, three demarcation points dividing opposite domains (i.e., $w_\lambda = 1$ and $w_\lambda = -1$) exist in Fig. S6C, corresponding to the Bloch points with delocalized OBC eigenmodes. Note that these Bloch points and the corresponding bipolar NHSE originate from the inherent high non-locality of long-range lattices, rather than the trivial case, $\lambda = 1$. In addition, despite that the OBC and PBC spectra in Fig. S6B possess intersection points (e.g., $\lambda = 0.03$), these points cannot separate domains with opposite winding numbers and thus are trivial and unable to constitute a Bloch point, since the latter is completely delocalized and separates the skin modes with opposite polarizations. In Fig. S6D, all SIBC and OBC eigenmodes are localized at the top boundary, consistent with $w_\lambda = -1$.

5. SIBC spectra and eigenstates

5.1. Non-reciprocal short-range lattices

For non-reciprocal short-range lattices with only the nearest-neighbor interactions shown in Fig. 1A, suppose that the infinite system is truncated at a certain horizontal position, e.g., $m = 0$. Then, a pair of semi-infinite lattices with a single boundary is formed. Without losing generality, let us consider the upper part ($m \geq 0$). Specifying $d_{n,m} \propto z^m \lambda^n$ with a complex wavenumber, $z \in \mathbb{C}$, the bulk equilibrium equation is exactly the eigen-equation of H, $\det\left[H(z) - \lambda\right] = 0$. The eigen-equation can be further simplified as $k_t - \lambda - \lambda^{-1} = z\left(k_2\lambda^{-1} + k_1\lambda\right) + z^{-1}\left(k_1\lambda^{-1} + k_2\lambda\right)$ with $k_t = 2\left(1 + k_1 + k_2\right)$. The preceding expression corresponds to a recursion relationship, and two eigen-solutions z_1 and z_2 can be solved for any reference eigenvalue, λ. In addition, the fixed ($m = 0$) and infinite ($m \to \infty$) boundary conditions specify that $d_{n,0} = 0$ and $d_{n,m\to\infty} = 0$, respectively, corresponding to the lower-SIBC edge states localized at the bottom boundary. A general solution is the superimposition of a pair of degenerate eigenmodes, i.e., $d_{n,m} = \lambda^n\left(c_1 z_1^m + c_2 z_2^m\right)$ with coefficients c_1 and c_2 determined by boundary conditions. From $d_{n,0} = 0$ we have $c_1 = -c_2$, which determines the profiles of SIBC edge states, whereas $d_{n,m\to\infty} = 0$ imposes that $|z_1| < 1$ and $|z_2| < 1$ simultaneously, from which we obtain the corresponding SIBC spectrum. A similar procedure can be employed for the lower part ($m \leq 0$), for which the SIBC spectrum is restricted within the region with $|z_1| > 1$ and $|z_2| > 1$. Results for different stiffness parameters are shown in Fig. 2A and B.

5.2. Domain wall between two lattices

A pair of semi-infinite short-range lattices with stiffnesses $(k_1, k_2) = (0.01, 0.03)$ and $(0.03, 0.01)$ connects each other at $m = 0$ and forms a domain wall, as shown in Fig. 2C. Inspired by the Hermitian case (*63*), for the lower half part of the lattice with $m < 0$, we specify that $d_{n,m} \propto z^m \lambda^n$, while the imposed displacement profile of the upper half part with $m > 0$ is either symmetrically ($d_{n,m} = d_{n,-m}$) or anti-symmetrically ($d_{n,m} = -d_{n,-m}$) distributed with respect to the lower part. The bulk equilibrium equations for the two sectors are consistent since they are mirror images of each other. For symmetric mode, the equilibrium condition of domain wall yields that $(k_t - \lambda - \lambda^{-1}) d_{n,0} = (k_2 \lambda^{-1} + k_1 \lambda)(d_{n,1} + d_{n,-1})$, from which the SIBC edge states profiles, i.e., $(k_t - \lambda - \lambda^{-1} - 2z_1 k_2 \lambda^{-1} - 2z_1 k_1 \lambda) c_1 + (k_t - \lambda - \lambda^{-1} - 2z_2 k_2 \lambda^{-1} - 2z_2 k_1 \lambda) c_2 = 0$, can be obtained. The SIBC spectrum can be solved according to the remaining two boundary conditions, $d_{n,m \to \pm\infty} = 0$, from which we have $|z_{1,2}| > 1$. Because $d_{n,0} = 0$ is always satisfied for the anti-symmetric mode with arbitrary n, edge states take the form of $c_1 = -c_2$ and the spectrum is restricted within the region $|z_{1,2}| > 1$. Consequently, regardless of the deformation modes, the SIBC spectrum of the domain wall is confined within the upper PBC branch of its constituent part, as marked by the cyan area in Fig. 2B.

5.3. Non-reciprocal long-range lattices

Consider the long-rang lattice shown in Fig. S6A truncated at $m = -1$, and take the upper half part as an illustrative example. The bulk equilibrium equation generally consists of four eigen-solutions, z_1, z_2, z_3 and z_4 for a reference λ. We sort them according to $|z_1| \le |z_2| \le |z_3| \le |z_4|$. A pair of fixed boundary conditions require that $d_{n,0} = d_{n,-1} = 0$. With the superposition solution $d_{n,m} = \lambda^n \sum_{i=1}^{4} c_i z_i^m$, we have

$$d_{n,0} = c_1 + c_2 + c_3 + c_4 = 0 \tag{S12}$$

$$d_{n,-1} = c_1 z_1^{-1} + c_2 z_2^{-1} + c_3 z_3^{-1} + c_4 z_4^{-1} = 0 \tag{S13}$$

The above two formulas are linear homogeneous equations about unknown coefficients c_i that can be determined by the boundary conditions at infinity (i.e., $d_{n,m \to \infty} = 0$), for which the only wavenumbers with moduli less than one are physically admissible. Consequently, the following five conditions can be distinguished:

(i) When all four eigen-solutions have moduli less than one, the whole set of coefficients is admissible. Combining Eqs. (S12) and (S13), we have

$$c_3 = \frac{z_1^{-1} - z_4^{-1}}{z_4^{-1} - z_3^{-1}} c_1 + \frac{z_2^{-1} - z_4^{-1}}{z_4^{-1} - z_3^{-1}} c_2 \qquad c_4 = \frac{z_3^{-1} - z_1^{-1}}{z_4^{-1} - z_3^{-1}} c_1 + \frac{z_3^{-1} - z_2^{-1}}{z_4^{-1} - z_3^{-1}} c_2 \tag{S14}$$

and the formation of the SIBC edge states is

$$d_{n,m}=\lambda^n\left[c_1\left(z_1^m+\frac{z_1^{-1}-z_4^{-1}}{z_4^{-1}-z_3^{-1}}z_3^m+\frac{z_3^{-1}-z_1^{-1}}{z_4^{-1}-z_3^{-1}}z_4^m\right)+c_2\left(z_2^m+\frac{z_2^{-1}-z_4^{-1}}{z_4^{-1}-z_3^{-1}}z_3^m+\frac{z_3^{-1}-z_2^{-1}}{z_4^{-1}-z_3^{-1}}z_4^m\right)\right] \quad \text{(S15)}$$

The fundamental solution of the above formula is spanned by two independent coefficients, c_1 and c_2, indicating the existence of two-fold degenerate SIBC edge states localized at the bottom boundary.

(ii) In the case when only $|z_4|>1$, we have $c_4=0$ and the formation of the SIBC edge states is

$$d_{n,m}=\lambda^n\left(\frac{z_3^{-1}-z_2^{-1}}{z_2^{-1}-z_1^{-1}}z_1^m+\frac{z_1^{-1}-z_3^{-1}}{z_2^{-1}-z_1^{-1}}z_2^m+z_3^m\right)c_3 \quad \text{(S16)}$$

which indicates that only a single SIBC edge state survives.

(iii) When both $|z_4|>1$ and $|z_3|>1$ are satisfied, we have $c_3=c_4=0$. From Eqs. (S12) and (S13), one can check that $z_1=z_2$ and $c_1=-c_2$, giving rise to a trivial solution, $d_{n,m}=\lambda^n\left(c_1z_1^m+c_2z_2^m\right)=0$.

(iv) If only $|z_1|<1$, we have $c_2=c_3=c_4=0$ and the solution is trivial.

(v) If all four eigen-solutions have moduli larger than one, the solution is trivial.

To sum up, only cases (i) and (ii) possess non-trivial SIBC bottom-edge states. The former has two-fold degenerate edge states while the latter has a single one. The SIBC spectrum and the corresponding top-localized edge states for the lower part can be solved in a similar manner. The results for different stiffness parameters are shown in Fig. S6B to D.

6. OBC spectra and eigenstates

6.1. Non-reciprocal short-range lattices

For a short-range lattice with finite size, $M<\infty$, the equilibrium equation for an internal column n with $n>0$ can be written in a matrix form as

$$[K]\{d_n\}-[K_1]\{d_{n-1}\}-[K_1]^T\{d_{n+1}\}=0 \quad \text{(S17)}$$

where $\{d_n\}$ is the displacement vector of the nth column, and the stiffness matrixes are defined as

$$[K]=\begin{bmatrix}k_a &&&& \\ & k_t &&& \\ && \cdots && \\ &&& k_t & \\ &&&& k_a\end{bmatrix}_{M\times M} \qquad [K_1]=\begin{bmatrix}1 & k_2 &&& k_b \\ k_1 & 1 & k_2 && \\ & k_1 & \cdots & k_2 & \\ && k_1 & 1 & k_2 \\ k_c &&& k_1 & 1\end{bmatrix}_{M\times M} \quad \text{(S18)}$$

where k_a and k_b are dominated by boundary conditions. For example, the cylindrical topology of PBC requires that $k_a=k_t$, $k_b=k_1$ and $k_c=k_2$, while for an open boundary system with fixed-fixed or free-free boundary conditions, we have $k_a=k_t$ and $k_b=k_c=0$ or $k_a=2+k_1+k_2$

and $k_b = k_c = 0$, respectively. The fixed-fixed boundary condition implies that the top and bottom edges of the finite system are fully hinged, while the system is unconstrained under the free-free boundary condition. Assuming $d_{n,m} = \lambda^n d_{0,m}$ and substituting it into Eq. (S17), we have

$$\left(\lambda^2 [K_1]^T - \lambda[K] + [K_1]\right)\{d_0\} = 0 \qquad \text{(S19)}$$

where $\lambda \neq 0$ is assumed. Otherwise, if $\lambda = 0$, one has $[K_1]\{d_0\} = 0$ from Eq. (S19), indicating a trivial solution owing to the non-singularity of $[K_1]$ under OBC. A nontrivial solution can be obtained only if the determinant of the coefficient matrix in Eq. (S19) is zero, $\det\left(\lambda^2 [K_1]^T - \lambda[K] + [K_1]\right) = 0$. Then, the OBC spectrum is determined by solving this 2*M*th polynomial about λ, and the corresponding eigenstates can be resolved by resubstituting λ into Eq. (S19). Note that $[K]$ is a symmetric matrix. Thus, from Eq. (S19) we also have $\det\left(\lambda^2 [K_1] - \lambda[K] + [K_1]^T\right) = 0$, indicating that the lattices with switched stiffnesses (k_1, k_2) and (k_2, k_1) share the same spectrum, both for the PBC and OBC. Intuitively, this is because these two lattices are mirror images of each other with respect to the *n* axis, and they have identical decay factors and reversed eigenstates, i.e., oppositely localized skin modes. The spectrum and eigenstates of the non-reciprocal short-range lattices under OBC are shown in Fig. 2.

6.2. Domain wall between two lattices

In analogy to text 6.1, the equilibrium equation for the domain wall under OBC can be expressed as Eq. (S19), where the stiffness matrixes are given by

$$[K] = \begin{bmatrix} k_a & & & & & & \\ & k_t & & & & & \\ & & k_t & & & & \\ & & & \cdots & & & \\ & & & & k_t & & \\ & & & & & k_t & \\ & & & & & & k_a \end{bmatrix}_{M\times M} \qquad [K_1] = \begin{bmatrix} 1 & k_1 & & & & & \\ k_2 & 1 & k_1 & & & & \\ & k_2 & 1 & k_1 & & & \\ & & & \cdots & & & \\ & & & k_1 & 1 & k_2 & \\ & & & & k_1 & 1 & k_2 \\ & & & & & k_1 & 1 \end{bmatrix}_{M\times M} \qquad \text{(S20)}$$

with the size M denoting the total number of nodes along the vertical direction ($M/2$ for each part). The two constituent parts share an identical PBC spectrum (Fig. 2B), while the spectral orientations are opposite. For an OBC eigenvalue lying on the left side of the Bloch point, its effective winding number with respect to the lower or upper part is 1 or -1, respectively, implying that the associated eigenstate is the skin mode localized at the two open boundaries. Whereas the corresponding skin mode is localized on the domain wall for eigenvalue lying on the right side of the Bloch point. The numerical solution shown in Fig. 2F confirms our analysis.

6.3. Non-reciprocal long-range lattices

Owing to the high non-locality of the long-range lattices shown in Fig. S6A, the open boundary conditions for a finite system are generally complicated, for which we only focus on the fixed-fixed or free-free boundary conditions in this context. The stiffness matrixes of a finite long-rang lattice are given by

$$[K]=\begin{bmatrix} k_a & & & & & & \\ & k_a^{'} & & & & & \\ & & k_t & & & & \\ & & & \cdots & & & \\ & & & & k_t & & \\ & & & & & k_a^{'} & \\ & & & & & & k_a \end{bmatrix}_{M\times M} \quad [K_1]=\begin{bmatrix} 1 & k_2 & k_4 & & & & \\ k_1 & 1 & k_2 & k_4 & & & \\ k_3 & k_1 & 1 & k_2 & k_4 & & \\ & & & \cdots & & & \\ & & k_3 & k_1 & 1 & k_2 & k_4 \\ & & & k_3 & k_1 & 1 & k_2 \\ & & & & k_3 & k_1 & 1 \end{bmatrix}_{M\times M} \tag{S21}$$

For the fixed-fixed boundary condition, we have $d_{n,-1}=d_{n,0}=d_{n,L+1}=d_{n,L+2}=0$ and $k_a=k_a^{'}=k_t$, whereas we have $k_a=2+k_1+k_2+k_3+k_4$ and $k_a^{'}=2+2k_1+2k_2+k_3+k_4$ for the free-free boundary condition. It can be seen that the difference of the OBC spectra between the two boundary conditions is negligible in a large system (the spectra under the free-free boundary condition are marked by black dots in Fig. S6B to D, with the size $M=40$, and we find that they are nearly identical to the OBC spectra under the fixed-fixed boundary condition), as also elucidated in text 8. Both spectra are constrained within a continuous bulk band and can be obtained analytically, as detailed in text 7.

7. Generalized Brillouin zone of decay spectrum

First proposed in Ref (*30*) for a 1D non-Hermitian Su-Schrieffer-Heeger (SSH) model, the GBZ has been widely shown to be a powerful method to characterize the non-Hermitian topology in various physical images (*30*, *64*, *65*), which restores the BEC even in the presence of non-Hermiticity (non-reciprocity). On one hand, the GBZ predicts the OBC spectrum and eigenstates at the thermodynamic limit ($M\to\infty$), while on the other hand, the topologically protected edge states can also be predicted based on the non-Bloch topological invariants defined in the GBZ (*30*). The basic idea of the GBZ is to superimpose the degenerate eigenstates of an OBC eigenvalue and select the appropriate coefficients to form the standing wave inside the finite structure. To be specific, the bulk equilibrium equation under the OBC is identical to that of the PBC, which can be expressed as $f(z,\lambda)=\det[H(z)-\lambda]=0$ with a complex wavenumber, $z\in\mathbb{C}$. From the former condition, $2g$ complex wavenumbers can be solved for a given λ and are ordered in accordance with their moduli, $|z_1(\lambda)|\le|z_2(\lambda)|\le\cdots|z_{2g}(\lambda)|$, where g denotes the range of interactions. For instance, $g=1$ for short-range lattices and $g=2$ for long-range lattices. The number of coefficients c_i is $2g$ in the superimposition modal $d_{n,m}=\lambda^n\sum_{i=1}^{2g}c_i z_i^m$ and can be determined by $2g$ independent boundary conditions, with g conditions for each boundary. At the thermodynamic limit, there should be at least a pair of complex wavenumbers satisfying $|z_i(\lambda)|=|z_j(\lambda)|$, so as to form a standing wave inside the

structure. Further analysis shows that only a pair of middle roots with equal lengths, i.e., $|z_g(\lambda)| = |z_{g+1}(\lambda)|$, are appropriate candidates for the GBZ (*66*), which generally forms a closed loop deviating from the unit circle (BZ) on the complex plane. Each point located on the GBZ maps an OBC eigen-solution (i.e., eigenvalue and eigenstate) in real space, and by traversing the whole GBZ, we can obtain the continuous OBC spectrum as well as skin modes. Here, we utilize the auxiliary generalized Brillouin zone method (aGBZ) (*64*) to calculate the GBZ analytically. According to the GBZ equation, we can define that $z_g = e^{i\theta} z_{g+1}$, whose characteristic equations are $f(z,\lambda)=0$ and $f(ze^{i\theta},\lambda)=0$, respectively. The necessary and sufficient condition for the former two polynomials to share a common root is that the resultant (or Sylvester determinant) of the two is zero, i.e., $\mathrm{res}\left[f(z,\lambda), f(ze^{i\theta},\lambda)\right]=0$ (*64*). For a given θ, the corresponding $z(\theta)$ can be obtained from the former condition. Traversing $\theta \in [0, 2\pi)$, the set of all z and $ze^{i\theta}$ forms the aGBZ, from which the GBZ can be determined by selecting the roots satisfying $|z_g(\lambda)| = |z_{g+1}(\lambda)|$.

7.1. aGBZs and GBZs of short-range lattices

The characteristic equation of the short-range lattices is

$$f(z,\lambda) = \left(k_1 z^2 + z + k_2\right)\lambda^2 - 2z\left(1+k_1+k_2\right)\lambda + k_2 z^2 + z + k_1 = 0 \qquad \text{(S22)}$$

and the resultant with respect to $f(z,\lambda)$ and $f(ze^{i\theta},\lambda)$ is

$$\mathrm{res}\left[f(z,\lambda), f(ze^{i\theta},\lambda)\right] = \begin{vmatrix} k_1 z^2 + z + k_2 & -2z(1+k_1+k_2) & k_2 z^2 + z + k_1 & 0 \\ 0 & k_1 z^2 + z + k_2 & -2z(1+k_1+k_2) & k_2 z^2 + z + k_1 \\ k_1 z^2 e^{2i\theta} + ze^{i\theta} + k_2 & -2ze^{i\theta}(1+k_1+k_2) & k_2 z^2 e^{2i\theta} + ze^{i\theta} + k_1 & 0 \\ 0 & k_1 z^2 e^{2i\theta} + ze^{i\theta} + k_2 & -2ze^{i\theta}(1+k_1+k_2) & k_2 z^2 e^{2i\theta} + ze^{i\theta} + k_1 \end{vmatrix} \qquad \text{(S23)}$$

The relationship between z and θ is determined by $\mathrm{res}\left[f(z,\lambda), f(ze^{i\theta},\lambda)\right]=0$. The weak non-locality of short-range lattices implies the coincidence of aGBZ and GBZ. In this way, the GBZ can be completely determined by traversing θ from 0 to 2π. Figure S7A and B depicts the GBZs of two distinct phases, where the light blue and blue solid lines represent the GBZs for the upper (GBZ-u) and lower (GBZ-l) branches, respectively, while the red dashed line represents the BZ.

These non-unitized GBZs contain crucial information for an open boundary system. (i) The two GBZs are located either inside or outside the BZ, indicating that the OBC eigenstates are no longer extended Bloch waves with real wavenumbers and are instead the skin modes localized at either the bottom or top boundary. (ii) The two GBZs and BZ intersect at $z=1$, and the corresponding OBC eigenvalue of the intersection lies on the PBC spectrum, with its eigenstate fully delocalized, corresponding to the Bloch point induced by the rigid-body displacement. In particular, for the lattice with $(k_1,k_2)=(0.01,0.03)$, the farther it is away from the Bloch point,

the more the modulus $|z|$ deviates from the unit value (Fig. S7A), which signifies enhanced localization of the skin mode, consistent with the numerical results of a finite model discussed in the main text (Fig. 2E). (iii) The two GBZs are not independent of each other in principle. One can check that for any ray starting from the origin, the product of the moduli of two intersection points (red dots in Fig. S7A and B) between the ray and two GBZs is exactly equal to one, which directly embodies the overall reciprocity protected by inversion symmetry in real space (see text 7.3 for more details). The continuous OBC spectrum at the thermodynamic limit can be obtained by resubstituting the GBZs to Eq. (S22) and is consistent with the numerical results obtained from a finite model discussed above.

7.2. aGBZs and GBZs of long-range lattices

The characteristic equation of the long-range lattices is

$$\begin{aligned} f(z,\lambda) &= \left(k_3 z^4 + k_1 z^3 + z^2 + k_2 z + k_4\right)\lambda^2 \\ &\quad - 2z^2\left(1+k_1+k_2+k_3+k_4\right)\lambda + k_4 z^4 + k_2 z^3 + z^2 + k_1 z + k_3 = 0 \end{aligned} \tag{S24}$$

with the resultant about $f(z,\lambda)$ and $f(ze^{i\theta},\lambda)$ given by

$$\operatorname{res}\left[f(z,\lambda), f(ze^{i\theta},\lambda)\right] = \begin{vmatrix} k_3 z^4 + k_1 z^3 + z^2 + k_2 z + k_4 & -2z^2\left(1+k_1+k_2+k_3+k_4\right) & k_4 z^4 + k_2 z^3 + z^2 + k_1 z + k_3 & 0 \\ 0 & k_3 z^4 + k_1 z^3 + z^2 + k_2 z + k_4 & -2z^2\left(1+k_1+k_2+k_3+k_4\right) & k_4 z^4 + k_2 z^3 + z^2 + k_1 z + k_3 \\ k_3 z^4 e^{4i\theta} + k_1 z^3 e^{3i\theta} + z^2 e^{2i\theta} + k_2 z e^{i\theta} + k_4 & -2z^2 e^{2i\theta}\left(1+k_1+k_2+k_3+k_4\right) & k_4 z^4 e^{4i\theta} + k_2 z^3 e^{3i\theta} + z^2 e^{2i\theta} + k_1 z e^{i\theta} + k_3 & 0 \\ 0 & k_3 z^4 e^{4i\theta} + k_1 z^3 e^{3i\theta} + z^2 e^{2i\theta} + k_2 z e^{i\theta} + k_4 & -2z^2 e^{2i\theta}\left(1+k_1+k_2+k_3+k_4\right) & k_4 z^4 e^{4i\theta} + k_2 z^3 e^{3i\theta} + z^2 e^{2i\theta} + k_1 z e^{i\theta} + k_3 \end{vmatrix} \tag{S25}$$

Because we have $g=2$ for the long-rang lattices, four complex wavenumbers can be solved from Eq. (S24) for a given λ, whereas only the middle two roots with $|z_2|=|z_3|$ form the GBZ. The aGBZs (black solid line) and their corresponding GBZs (blue and light blue dots) of three typical cases are shown in Fig. S7C to E, where the two are no longer consistent with each other due to the high non-locality of the long-range lattices.

It can be seen that the GBZ-l is completely localized within or without the BZ from Fig. S7C or E, indicating the corresponding skin modes are either bottom- or top-localized, whereas from Fig. S7D, there are additional intersections between the GBZ-u, GBZ-l and BZ, rather than the trivial case, $z=1$, corresponding to the Bloch points originating from the inherent high non-locality of the long-range lattices. For example, the OBC eigenvalue at the intersection point $0.56 \pm 0.84i$ is $\lambda = 0.102$, a real Bloch point separating the bipolar skin modes (Fig. S6F). Another two Bloch points with complex eigenvalues, $\lambda = -0.15 \pm 0.17i$, are not shown in Fig. S6F owing to the complexity of eigenvalues and eigenstates.

7.3. Symmetry protected GBZs of the static Rayleigh model and the $\mathbb{Z}_2$ skin effect

As mentioned above, the reciprocal skin effect in our model resembles the $\mathbb{Z}_2$ skin effect in quantum mechanics. In momentum space, both the two models have GBZs formed by a pair of reciprocal loops, i.e., z and z^{-1}, corresponding to the reciprocal skin effects localized on opposite boundaries.

For the $\mathbb{Z}_2$ skin effect, the Hamiltonian is protected by the anomalous time reverse symmetry ($\mathrm{TRS}^{\dagger}$), $U_T H^T(k) U_T^{-1} = H(-k)$, with a unitary matrix U_T and $U_T U_T^* = -1$ (*26*, *58*, *67*). The overall spectral winding number is trivialized by $\mathrm{TRS}^{\dagger}$ because the paired PBC spectra coincide and orient in opposite directions. In terms of $\mathrm{TRS}^{\dagger}$, the characteristic equation satisfies (*68*)

$$\begin{aligned} f(z,E) &= \det\left(U_T H^T\left(z^{-1}\right)U_T^{-1} - E\right) = \det\left(H^T\left(z^{-1}\right) - E\right) \\ &= \det\left(H\left(z^{-1}\right) - E\right) = f\left(z^{-1}, E\right) \end{aligned} \quad \text{(S26)}$$

As a result, the GBZ consists of two reciprocal loops of $z_1 = z_2^{-1}$, with the OBC eigenvalues being doubly degenerate and their eigenstates localized on opposite boundaries to form a Kramers pair (*58*).

As for the reciprocity of the skin effect in our model, it originates from the space inversion symmetry, instead of the nonspatial $\mathrm{TRS}^{\dagger}$. The corresponding Hamiltonian respects the symmetry $\sigma_x H(q) \sigma_x = H^{-1}(-q)$ with σ_x the Pauli matrix, for which the decay factors and wavenumbers are in the form of a reciprocal pair, $\lambda(-q) = \lambda^{-1}(q)$ (or equivalently, $\eta(-q) = -\eta(q)$).

In the momentum space, the inversion symmetry manifests itself from the GBZ. The characteristic equation can be simplified as

$$\begin{aligned} f(z,\lambda) &= \det\left(\lambda H(z)\left(\lambda^{-1} - H^{-1}(z)\right)\right) = \det\left(\lambda H(z)\right)\det\left(\lambda^{-1} - H^{-1}(z)\right) \\ &= \det\left(\lambda H(z)\right)\det\left(\lambda^{-1} - \sigma_x H\left(z^{-1}\right)\sigma_x\right) \\ &= \det\left(\lambda H(z)\right)\det\left(\lambda^{-1} - H\left(z^{-1}\right)\right) = \det\left(\lambda H(z)\right) f\left(z^{-1}, \lambda^{-1}\right) \end{aligned} \quad \text{(S27)}$$

where, in general, $\det\left(\lambda H(z)\right)$ is nonzero. The above condition yields $f(z,\lambda) = f\left(z^{-1}, \lambda^{-1}\right) = 0$, indicating that the space inversion state $\left(z^{-1}, \lambda^{-1}\right)$ is also an eigenstate of the original system. Hence, both the wavenumber and decay factor form a reciprocal pair. According to the space inversion, a skin mode localized on one boundary that has a non-Bloch wavenumber z and a decay factor of $|\lambda| < 1$ (i.e., a static Rayleigh mode applied on the left boundary), can always be transmitted to another boundary, accompanied by a reversed wavenumber z^{-1} and decay factor $\left|\lambda^{-1}\right| > 1$ (corresponding to the right-applied static Rayleigh mode). The reciprocal pair is delocalized at the "inversion invariant" eigenvalue, $\lambda = 1$, namely, the rigid-body displacement

at the Bloch point. As a result, the reciprocal skin mode pairs in our model have different decay factors λ and λ^{-1} and are not degenerate while the $\mathbb{Z}_2$ skin effect is degenerate, sharing the same energy E (*26*).

8. Non-Bloch BEC under different boundary conditions and local defects

Despite the eigen-spectrum of a non-Hermitian system is sensitive to boundary conditions, e.g., PBC and OBC, whereas the OBC spectrum and skin modes are inert to the form of OBCs, because the skin modes are actually the bulk physics. For instance, the OBC spectrum captured by a discrete model with a free-free boundary condition is consistent with the continuous bands derived from the GBZ, for which the prerequisite boundary condition is actually fixed-fixed (*66*). This perspective also holds for the fixed-free boundary condition. Figure S8A shows the OBC spectra of a finite short-range lattice with $(k_1, k_2) = (0.01, 0.03)$ and various model sizes under three OBCs. Although the eigenvalues of different OBCs are generally different, they are constrained within the continuous band extracted from the GBZ (blue region). Moreover, the deviation of the eigenvalues between different OBCs is almost negligible with sufficiently large system size. Figure S8B and C shows the displacement distributions under the fixed-fixed and fixed-free boundary conditions, respectively, with the size being $M = 40$. Comparing the results with those of the free-free boundary condition shown in Fig. 2E, we find that the non-Bloch BEC remains valid regardless of the specific form of OBC, i.e., the OBC eigenvalues with moduli less or larger than one correspond to the bottom- or top-localized skin modes, while the states adjacent to the Bloch are almost delocalized.

Moreover, the OBC spectrum and skin modes seem to be insensitive to moderate structural defects, which may be deemed to be topologically protected by the intrinsic point-gap topology (*26*). As an example, we construct a short-range lattice sample with size $M = 20$ and $(k_1, k_2) = (0.01, 0.03)$ while gradually cut down the horizontal stiffnesses of the bottom end (i.e., $m = 1$) from the unit value to zero. The boundary condition is set as free-free. The OBC spectrum as well as the displacement distribution of certain branches are numerically calculated with respect to the defect strength, δ, which is defined as the reduction of horizontal stiffnesses at the bottom end.

Figure S8D shows the evolution of OBC eigenvalues as a function of δ, and we find that the distortion of the bulk OBC spectra (black lines) induced by structural defects is nearly negligible when δ is small, indicating the topological robustness. However, when the structural defect is large enough, e.g., $\delta > 0.56$, the highest and lowest branches (purple and blue lines) may jump out of the continuous band (blue region) and penetrate to the bulk gap (gray region). Figure S8E and F respectively shows the displacement distributions with respect to δ of two anomalous branches, from which we find an unexcepted phenomenon: the skin mode of the highest branch, which is originally top-localized ($m \to M$), migrates to the bottom end ($m \to 0$) when δ exceed the critical value, 0.56 (Fig. S8F), while the concentration strength of the bottom-localized lowest branch is enhanced (Fig. S8E), indicating the broken of the inversion symmetry. This local defect state is indued by the Anderson localization. Moreover, both branches tend to be top-localized with sufficient strong defects, e.g., $\delta > 0.93$ for the lowest branch and $\delta > 0.86$ for the highest branch. The underlying mechanism may stem from the competition between the structural rigidity and the nodal equilibrium condition. This can be understood by noting that the weakness of the horizontal bar in bottom end renders it more floppy and is able to concentrate intensive deformation compared with the top end, while the

bottom nodes can no longer be balanced once the horizontal interaction is almost absent. Hence, the deformation can only be concentrated at the top end whose rigidity is undisturbed.

9. Other topological effects in static Rayleigh model

In addition to the non-Hermitian topological phase elucidated in the main text (i.e., the NHSE), our model is more general that can emulate various topological effects (either spectral winding topology or wavefunction-based topology) originated from wave dynamics, applying both for Hermitian and non-Hermitian systems. Two additional prototypical examples of the topological phenomena in our static model are given in the following. From the first model we show a novel class of skin effect that has never been observed in a classical mechanical system. In the second model, an intriguing topological effect dubbed as the non-Hermitian morphing is demonstrated theoretically and experimentally in our conservative system, based on a purely passive modulation of the lattice configuration.

9.1. Critical non-Hermitian skin effect

The critical non-Hermitian skin effect (CNHSE) is a new class of skin effect with quantum criticality that have different topological states scaling with system size (*49*, *50*, *69–71*). Beyond a critical system size, the skin effect becomes scale-free. This anomalous localization differs from the conventional edge state with a fixed decay length. So far, CNHSE has only been proposed in quantum models such as a coupled HN chain (*50*) or coupled SSH chain (*71*), and none has been found in the classical wave systems. Here we demonstrate the CNHSE in our static model. Consider a bilayer short-range lattice with switched diagonal stiffnesses, i.e., (k_1, k_2) and (k_2, k_1) for the bottom and upper layers, respectively. The interlayer coupling is denoted by Δ, as shown in Fig. S9A. A unit cell contains two nodes, one from the bottom layer and the other from the top layer. The governing equations of the unit cell can be formulated as

$$\begin{cases} d_{n-1,m}^{(1)} + d_{n+1,m}^{(1)} + k_1\left(d_{n+1,m+1}^{(1)} + d_{n-1,m-1}^{(1)}\right) + k_2\left(d_{n-1,m+1}^{(1)} + d_{n+1,m-1}^{(1)}\right) + \Delta d_{n,m}^{(2)} = k_t d_{n,m}^{(1)} \\ d_{n-1,m}^{(2)} + d_{n+1,m}^{(2)} + k_2\left(d_{n+1,m+1}^{(2)} + d_{n-1,m-1}^{(2)}\right) + k_1\left(d_{n-1,m+1}^{(2)} + d_{n+1,m-1}^{(2)}\right) + \Delta d_{n,m}^{(1)} = k_t d_{n,m}^{(2)} \end{cases} \tag{S28}$$

where the total stiffness is $k_t = 2(1+k_1+k_2)+\Delta$. Supposing a static Rayleigh solution $d_{n,m}^{(i)} = \psi_i e^{iqm}\lambda^n$ and substituting it into Eq. (S28), we have

$$\begin{bmatrix} u + e^{iq}\xi_1 + e^{-iq}\xi_2 - k_t & \Delta \\ \Delta & u + e^{iq}\xi_2 + e^{-iq}\xi_1 - k_t \end{bmatrix} \begin{Bmatrix} \psi_1 \\ \psi_2 \end{Bmatrix} = 0 \tag{S29}$$

where $\xi_1 = k_1\lambda + k_2\lambda^{-1}$ and $\xi_2 = k_2\lambda + k_1\lambda^{-1}$, respectively. The decay spectrum can be obtained from a zero determinant in Eq. (S29). Because the two layers have switched stiffnesses, they share a common PBC spectrum but opposite windings, as marked by the black line in Fig. S9B, with the stiffnesses being $(k_1, k_2) = (0.01, 0.03)$. The numerically calculated OBC spectrum under various system sizes is marked by the solid dots in Fig. S9B. A remarkable feature of the OBC spectrum is the size dependence, even with an infinitesimal coupling strength, e.g., $\Delta = 10^{-3}$ in Fig. S9. The OBC spectrum, which is originally constrained at the real axis,

approaches the PBC spectral loop when the system size is sufficiently large (e.g., $M = 100$), manifesting the strong coupling effect of the two layers. Such a feature contradicts the non-Bloch band theory, which argues that the continuous spectrum at the thermodynamic limit coincides with that of a finite lattice, as confirmed in text 8 for a single-layer short-range lattice. The discontinuous jump of the OBC spectrum at the thermodynamic limit, i.e., from the real axis to the PBC spectrum, can be triggered by any nonzero Δ, as already demonstrated in a coupled HN chain (*49*). This discontinuity also indicates the delocalization of the skin mode when $M \to \infty$, where the non-reciprocities of the two layers cancel out and the model is net-reciprocal with only extended bulk modes. In other words, it means that the decay length of the finite system is not fixed, but scales with the system size as $\kappa \sim 1/M$, for which the entire decay rate, defined as $\left|d_{n,M}^{(i)}\right| / \left|d_{n,1}^{(i)}\right|$, is independent of the system size. This is the scale-free feature of the critical system. Figure S9C shows the normalized OBC eigenstates of the two layers with various lattice sizes, where the eigenstates with the largest imaginary decay factor are presented. It can be seen that these skin modes share the same decay rate within the whole lattice. In fact, all these states converge to a fixed value when $m \to 0$ (top layer) and $m \to M$ (bottom layer), similar to the critical phenomenon observed in quantum mechanics (*49*, *50*). The simulation results shown in Fig. S9D has a relatively large deviation from the theoretical result compared with Fig. 4D, mainly due to the numerical error inherent in an ill-conditioned stiffness matrix, i.e., $\det[K] \to 0$ for the selected stiffness parameters.

The demonstrated CNHSE in the bilayer lattice is its first realization in a classical mechanical system. Moreover, the realization of the CNHSE in a coupled atomic chain requires fine-tuning of the non-reciprocity and the interchain coupling (*49*, *50*, *71*), while the experimental realization of the critical effect in our macroscopic model is much easier.

9.2. Non-Hermitian morphing of the topological zero mode

The topological zero mode (TZM) is localized at the boundary or interface in Hermitian systems. The skin effect provides a venue for morphing the profile of TZM, which has attracted much interest both theoretically (*72*, *73*) and experimentally (*48*). Non-Hermitian morphing of the TZM can be tailored straightforward in our passive static model, with only the lattice configuration being tuned while no external modulation needed, as shown in the following.

Note that the dimerized short-range lattice with two nodes per unit cell (*42*) does not possess the exact zero-decay factor TZM owing to the horizontal interactions that give rise to an additional diagonal term in the Hamiltonian (text 6.1). This diagonal term breaks the chiral symmetry that is necessary for a TZM. Therefore, we investigate a short-range lattice with neglected horizontal interactions, see Fig. 3A for the lattice configuration. Similar to the 1D dynamic model in Ref (48), our static Rayleigh model comprises two subparts separated by an interface (gray shaded area in Fig. 3A). The left part (red shaded area) is a non-Hermitian SSH model with spatially asymmetric couplings, where the TZM can be reconfigured from the skin effect. The right (blue shaded area) is a symmetric lattice with staggered diagonal couplings, emulating a Hermitian SSH model. The interface structure shown in Fig. 3A has 10 lattice nodes for every horizontal layer ($M = 10$), with 6 nodes for the non-Hermitian part and 4 nodes for the Hermitian part, respectively.

Consider three cases, i.e., (i) $k_1 = 1$ and $k_2 = 2$, (ii) $k_1 = k_2 = 1$, and (iii) $k_1 = 2$ and $k_2 = 1$, where the decay factors of their corresponding zero modes are 0.058717, 0.040920 and

0.071521, respectively. The nontrivial phase of the Hermitian SSH model lies in the parametric region with $k_1 > k_2$ (i.e., when the inter-cellular couplings are larger than the intra-cellular couplings, corresponding to case (iii)) where the TZM is localized on the right boundary, while for the non-Hermitian SSH model, the TZM can be pumped to the left boundary when $k_1 < k_2$ (i.e., case (i)). For case (ii) of $k_1 = k_2 = 1$, the system lies in the gapless phase with a delocalized bulk zero mode. As a result, by adjusting the lattice stiffness, the TZM can be morphed to different spatial configurations owing to the competition between the topological localization and the skin effect. The corresponding zero mode profiles of the three cases are shown in the left panels of Fig. 3B, showing a good agreement with the prediction.

We numerically and experimentally demonstrate the non-Hermitian morphing of TZM in our static model. The lattice size is $N \times M = 3 \times 12$ with the left ($m = 1$) and right ($m = 12$) nodes fixed as the boundary condition. The external load is applied on the top boundary ($n = 0$), and the TZM can be checked by measuring the deformation at the middle row ($n = 1$). The numerically measured decay factors, defined as the displacement ratios between $n = 1$ and $n = 0$, are 0.058719, 0.040919 and 0.071523, respectively, agreeing well with the theoretical results.

The experimental platform is the same as Fig. 4 but with the horizontal bars removed (i.e., $k_0 = 0$) to preserve the chiral symmetry. The right panels of Fig. 3B show the deformation plots of the three cases. It can be seen that the applied load is nearly blocked at the boundary with negligible response at $n = 1$, a strong evidence of the non-Hermitian morphed TZM in our passive system. The result indicates that, by tuning the lattice stiffness, zero modes can be morphed to different configurations, by which the loads with various profiles can be blocked with high deformation shielding inside the bulk.

10. Generality of the skin effect in static Rayleigh model

The skin effect is a general phenomenon for a lattice that tailors the effective non-reciprocity, with two prerequisites. First, noting that the static Rayleigh mode is a special solution of the equilibrium equation, this mode can be extracted from other modes by applying suitable boundary conditions, as clarified in our first experiment (Fig. 4). Second, the interlayer couplings along the spatial evolution direction can be adjusted to form a spatial asymmetric configuration. This condition tailors the effective nonreciprocity that is necessary for a skin mode, as achieved from the unequal diagonal stiffnesses for the short-range lattice. In general, we can combine the governing equation of the periodic lattice with its static Rayleigh solution, followed by reformulating them in an effective interaction form akin to the dynamic equation of a lower dimensional mass-spring model, as has been adopted to Eq. (1). This procedure is vital for a 2D static lattice to be mapped to a 1D mass-spring chain so that the non-Hermitian topological band theory is applicable. Once the effective interactions along different spatial directions (e.g., $k_{m\to m+1} = e^{-\eta}k_2 + e^{\eta}k_1$ and $k_{m+1\to m} = e^{-\eta}k_1 + e^{\eta}k_2$ for Eq. (1) in the main text) are asymmetric, the lattice is non-reciprocal in the dynamic sense and the skin effect emerges. It should be noted that our model only maps a class of non-Hermitian dynamic models with non-reciprocal couplings, owing to the discretization along the state evolution coordinate and the subsequent asymmetric coupling effects, while another kind of non-Hermiticity originated from the pure on-site gain/loss effect is unavailable in our energy conservative static system.

Because this mapping strategy is only relevant to the lattice configuration (so that the static Rayleigh solution is well defined) and is not related to internodal couplings, the effective non-reciprocity can be achieved in any long-range-coupling lattices with spatial asymmetric

interactions. This has already been demonstrated in text 4 for a long-range lattice with the next-nearest-neighbor couplings, where the effective non-reciprocity is achieved by either tuning the nearest- or next-nearest-couplings. These lattices can be employed to emulate a dynamic model chain with random non-reciprocal couplings. Moreover, it should be pointed out that the mapping is not restricted to the Bravais lattices with a single node. It is also applicable to compound lattices with multiple DoF within a unit cell, as demonstrated in text 9.2 for a dimerized short-range lattice with two nodes per unit cell.

The dimension mapping strategy between the 1D wave dynamic and 2D statics can be readily generalized to higher dimensions, e.g., 2D non-Hermitian dynamics emulated in 3D static lattices. Consider a 3D layered square lattice, as shown in Fig. 3C for the configuration of the associate cell, where the planar square lattice extends along the $n-m$ plane and is layered along the l axis. The lattice node is labeled by three indices, (n,m,l). The spatially asymmetric diagonal interlayer couplings are k_1 and k_2 (red and orange bars) in the $n-l$ plane, k_3 and k_4 (blue and cyan bars) in the $m-l$ plane, while the vertical interlayer coupling (gray bars) is unitized by convention (Fig. 3C). The intralayer stiffnesses are neglected. The lattice equilibrium equation is

$$\begin{aligned} &d_{n,m,l-1}+d_{n,m,l+1}+k_1\left(d_{n+1,m,l+1}+d_{n-1,m,l-1}\right)+k_2\left(d_{n-1,m,l+1}+d_{n+1,m,l-1}\right)\\ &+k_3\left(d_{n,m+1,l+1}+d_{n,m-1,l-1}\right)+k_4\left(d_{n,m+1,l-1}+d_{n,m-1,l+1}\right)=k_{\mathrm{t}}d_{n,m,l} \end{aligned} \tag{S30}$$

with the total stiffness $k_{\mathrm{t}}=2(1+k_1+k_2+k_3+k_4)$. Employing the static surface Rayleigh mode, i.e., $d_{n,m,l}=e^{i(q_n n+q_m m)}\lambda^l$ with q_n and q_m the components of wavevector and λ the decay factor, and substituting it into Eq. (S30), the decay spectrum can be obtained. The static Rayleigh mode can be excited by a delicate boundary load applied on the top surface, $l=0$. Based on the 2D (edge) Rayleigh mode illustrated above, it can be seen that the effective non-reciprocity along different spatial dimensions (i.e., $n-l$ plane and $m-l$ plane) can be independently actuated when either $k_1\neq k_2$ or $k_3\neq k_4$. Thus, the OBC eigenmodes are pumped to the boundaries or even the corners owing to the collective behavior of the skin effect.

The PBC spectra with different sets of stiffness parameters are shown in the left panels of Fig. 3D, where the nonzero spectral region signifies the skin effect (*74*). Only the applied top Rayleigh modes with $|\lambda|<1$ are shown for convenience. The mean amplitudes of all OBC bulk modes, defined as $\bar{d}_{n,m}=\frac{1}{MN}\sum_j^{NM}\left|d_{n,m}^j\right|^2$ with *j* the mode index and *N* and *M* the system size along the two planar axes (*NM* eigenmodes in total), are shown in the right panels of Fig. 3D. There are 10 lattice nodes along both the *n* and *m* axes (i.e., a square geometry). It is evident that the eigenmodes are localized at the edges or a corner, depending on which direction the associated reciprocity is broken. For example, when $k_3\neq k_4$ ($k_1\neq k_2$) and $k_1=k_2$ ($k_3=k_4$), the eigenmodes are pumped to the top/bottom (left/right) boundaries, while a higher-order corner mode is observed when $k_1\neq k_2$ and $k_3\neq k_4$ owing to the joint non-reciprocities along the two axes. Another set of oppositely localized skin modes with $|\lambda|>1$ is ensured by the 3D inversion symmetry (not shown here). These results are consistent with the higher-order non-Hermitian

topological effects observed in wave dynamic systems with designed non-reciprocal couplings (*32*, *75*), while our model is fully passive.

The above model provides further evidences for the generality of the effective non-reciprocity and the associated skin effect in our system. To sum up, our dimension mapping strategy, i.e., emulating the non-Hermitian topological effect in a higher-dimensional static system, is general, which is applicable to both 2D and 3D static lattices with random interactions and unit cell structures. Only the lattice configuration is tuned when tailoring the non-reciprocity in our model, which is of great advantages compared with the non-Hermitian wave dynamics with complicated active controls and gain/loss terms.

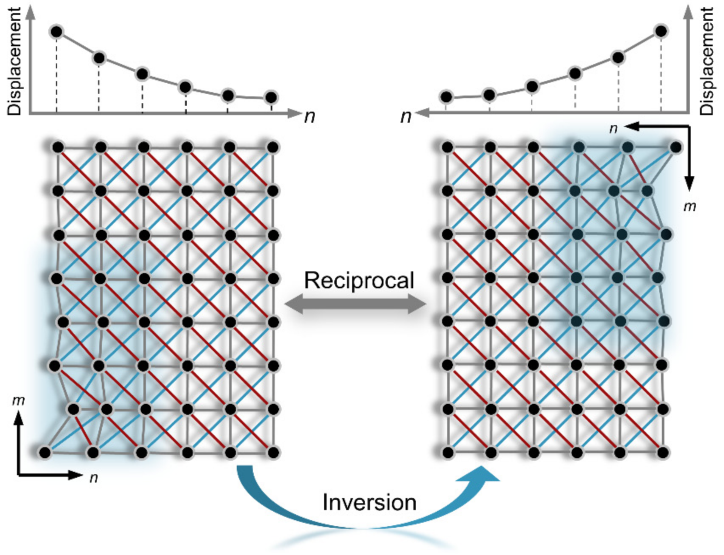

Fig. S1.

Overall reciprocity of the metamaterial. The system is invariant under spatial inversion. Hence, for any skin mode with a decay factor λ and localized at the bottom end, there must be a corresponding top-localized skin mode with λ^{-1}. The two form a reciprocal pair.

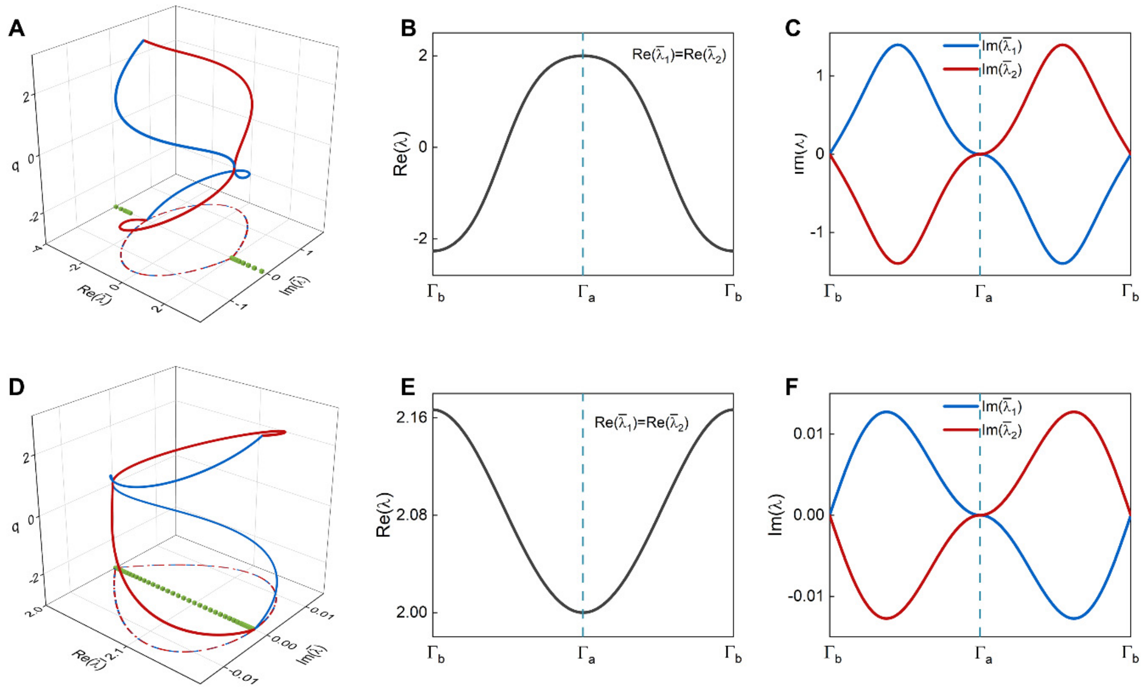

Fig. S2.

Spectrum of compound Hamiltonian. (**A** and **D**) Red and blue lines denote the PBC spectra of the two eigenvalues $\bar{\lambda}_1$ and $\bar{\lambda}_2$ for $\bar{H}(q)$ in the ($\mathrm{Re}(\bar{\lambda})$, $\mathrm{Im}(\bar{\lambda})$, q) space respectively, with the OBC spectra labeled by green cubes that are either localized without (A) or within (D) the PBC spectrum for distinct topological phases. (**B** and **E**) Real parts of two PBC spectra that are doubly degenerate in BZ. (**C** and **F**) Imaginary parts of two PBC spectra. The energy levels at opposite momenta are degenerate and form a parity pair. The two spectra coalesce at the inversion-invariant points Γ_a and Γ_b in the BZ. Stiffnesses are $(k_1, k_2) = (1, 15)$ for (A) to (C) and $(0.01, 0.03)$ for (D) to (F), respectively.

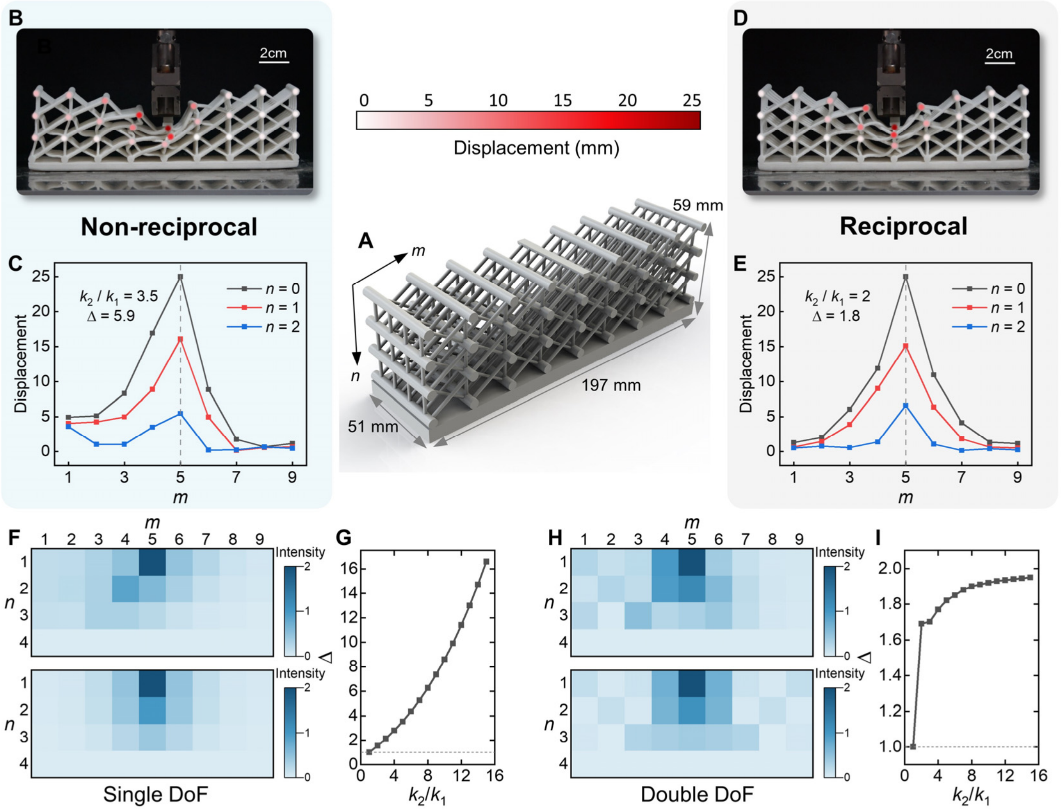

Fig. S3.

Frame-like lattices and the capability for load transfer. (**A**) Side view of the frame-like lattice with $N \times M = 3 \times 10$. (**B** and **D**) Deformation plots of the lattices under a compressive load, with the stiffness contrasts being $k_2/k_1 = 3.5$ (B) and 2 (D) respectively. (**C** and **E**) Corresponding nodal displacements of (B) and (D). (**F** and **H**) Simulated displacement fields of the frame-like lattices with a single (F) and double (H) DoF. Top and bottom panels denote the non-reciprocal ($k_2/k_1 = 6$) and reciprocal ($k_2/k_1 = 1$) lattices, respectively. (**G** and **I**) Measured deflection strength Δ for different stiffness contrasts in single (G) and double (I) DoF lattices.

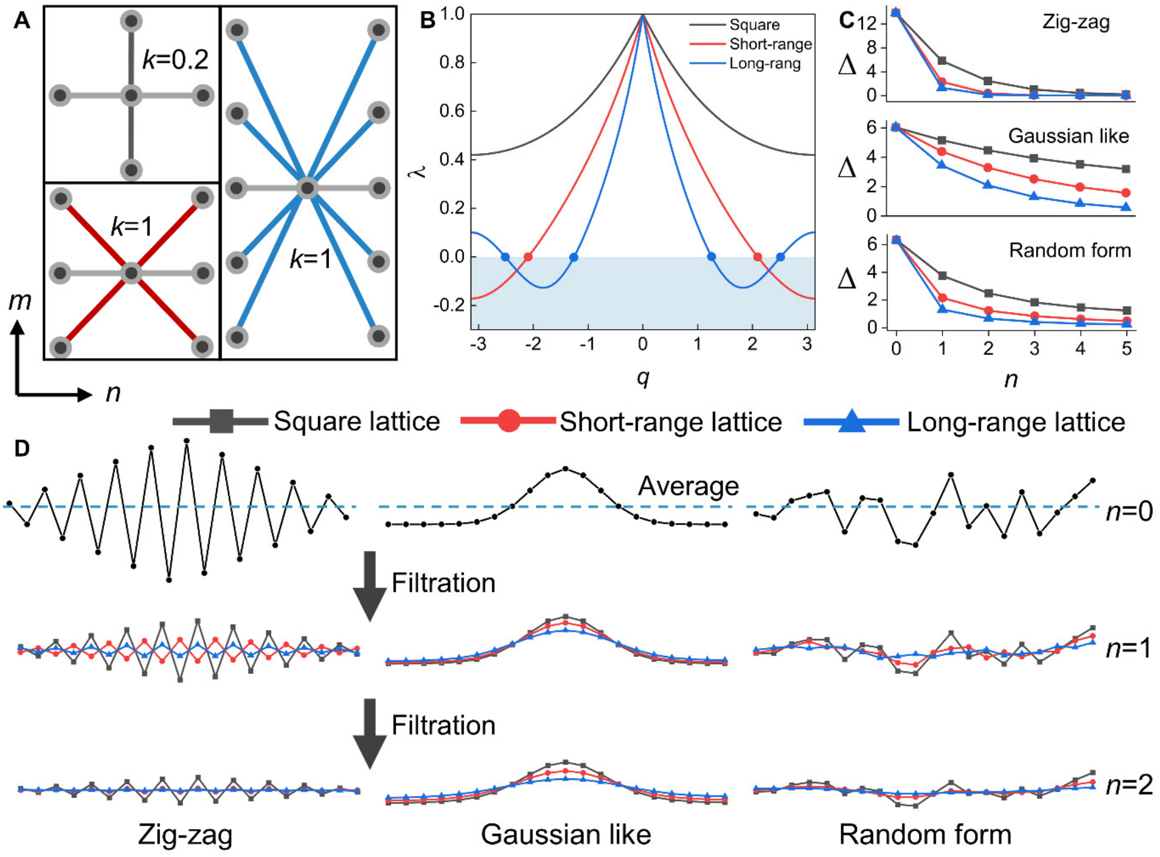

Fig. S4.

Reducing load roughness with zero mode. (**A**) Schematic of the associate cells of a square lattice, a short-range lattice, and a long-range lattice, respectively. The horizontal stiffness (gray bar) is unitized. (**B**) Decay spectra of the three lattices. The shaded area denotes the metamaterial region with a negative decay factor. (**C**) Roughness of the deformation of the three loading patterns, i.e., zig-zag, Gaussian, and random form. (**D**) Propagation of the deformation of the three loads from $n = 0$ to $n = 2$. Black, red, and blue lines denote the results of the square, short-range, and long-range lattices, respectively.

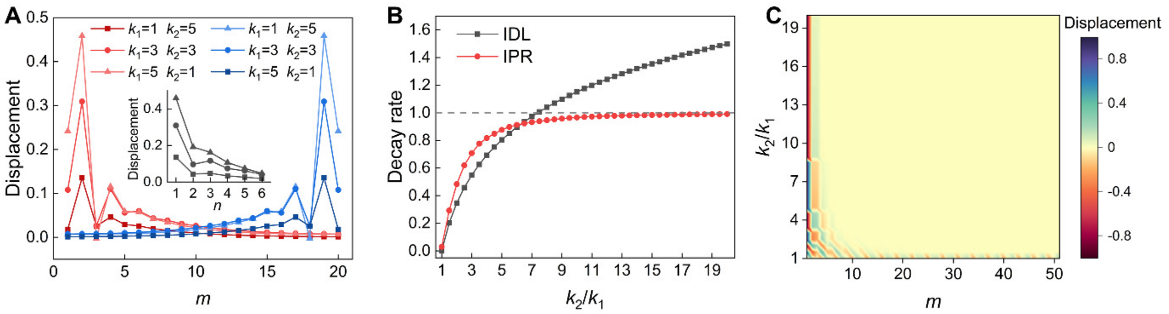

Fig. S5.

Combined effects of zero mode and skin effect. (**A**) Deformation blocking for the acentric point load applied at $m=1$ or $m=19$, respectively. (**B**) IDL and IPR of the zero skin modes. (**C**) Normalized displacement contours for the zero skin modes, with the size of the lattice being $M=51$.

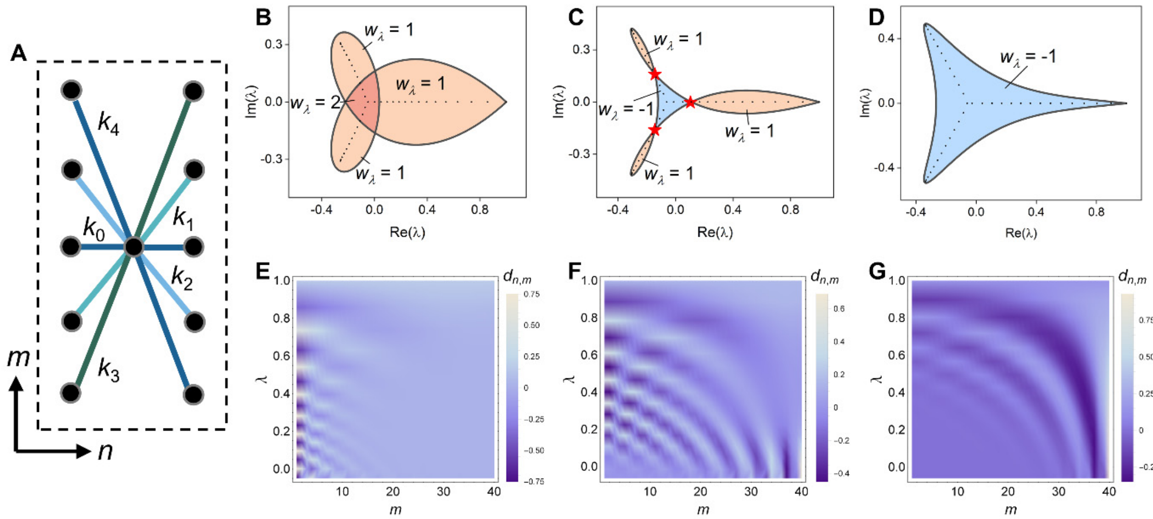

Fig. S6.

Decay spectra and skin modes of long-range lattices. (**A**) Schematic of the associate cell of a long-rang lattice with the next-nearest-neighbor interactions. (**B** to **D**) Decay spectra of the long-range lattice under different boundary conditions. The notations are the same as those in Fig. 2. (**E** to **G**) Corresponding skin mode profiles of (B) to (D), with the size $M = 40$. Stiffnesses are $(k_1, k_2, k_3, k_4) = (30,15,1,150)$ for (B) and (E), $(70,15,1,150)$ for (C) and (F), and $(140,15,1,150)$ for (D) and (G), respectively.

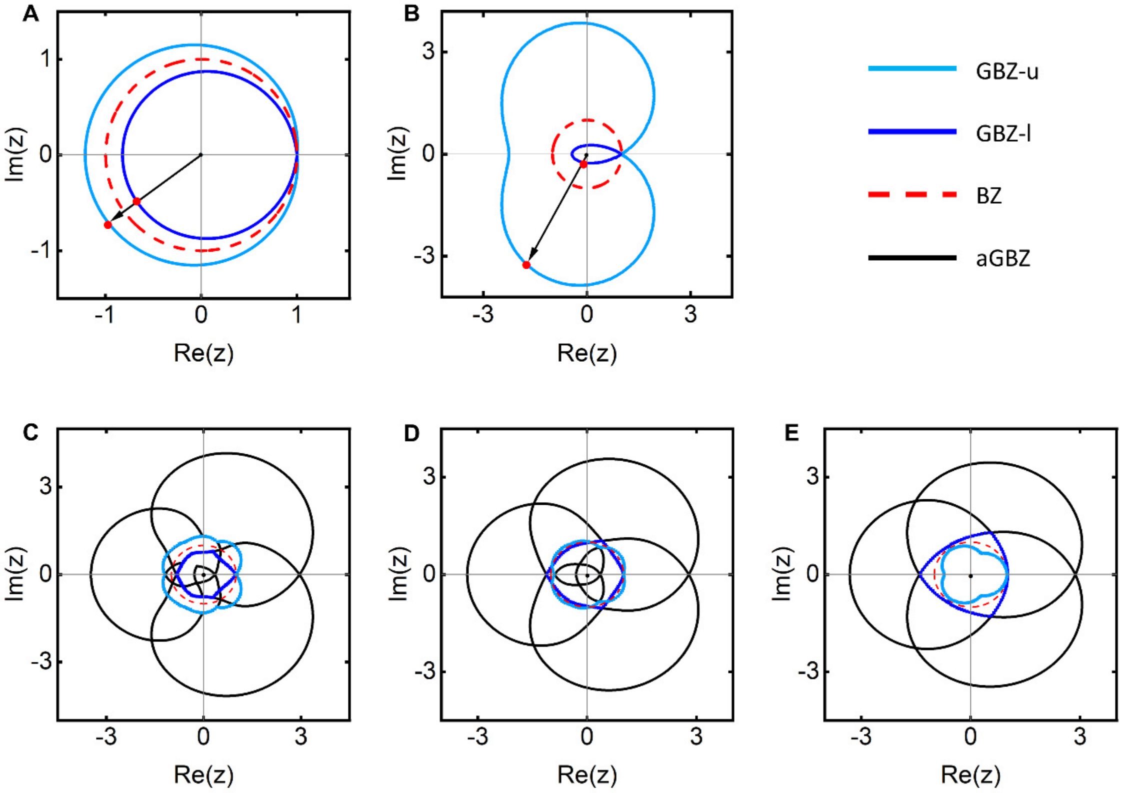

Fig. S7.

Generalized Brillouin zones and auxiliary generalized Brillouin zones. (**A** and **B**) GBZs of the short-range lattices with stiffnesses $(k_1, k_2) = (0.01, 0.03)$ (A) and $(1, 15)$ (B), respectively. The aGBZs coincide with GBZs owing to the low non-locality, $g = 1$. (**C** to **E**) GBZs and aGBZs of the long-range lattices with stiffnesses $(k_1, k_2, k_3, k_4) = (30, 15, 1, 150)$ (C), $(70, 15, 1, 150)$ (D) and $(140, 15, 1, 150)$ (E), respectively. Blue and light blue curves denote the GBZs of the lower and upper branches, while black solid and red dashed lines represent the aGBZs and BZ, respectively.

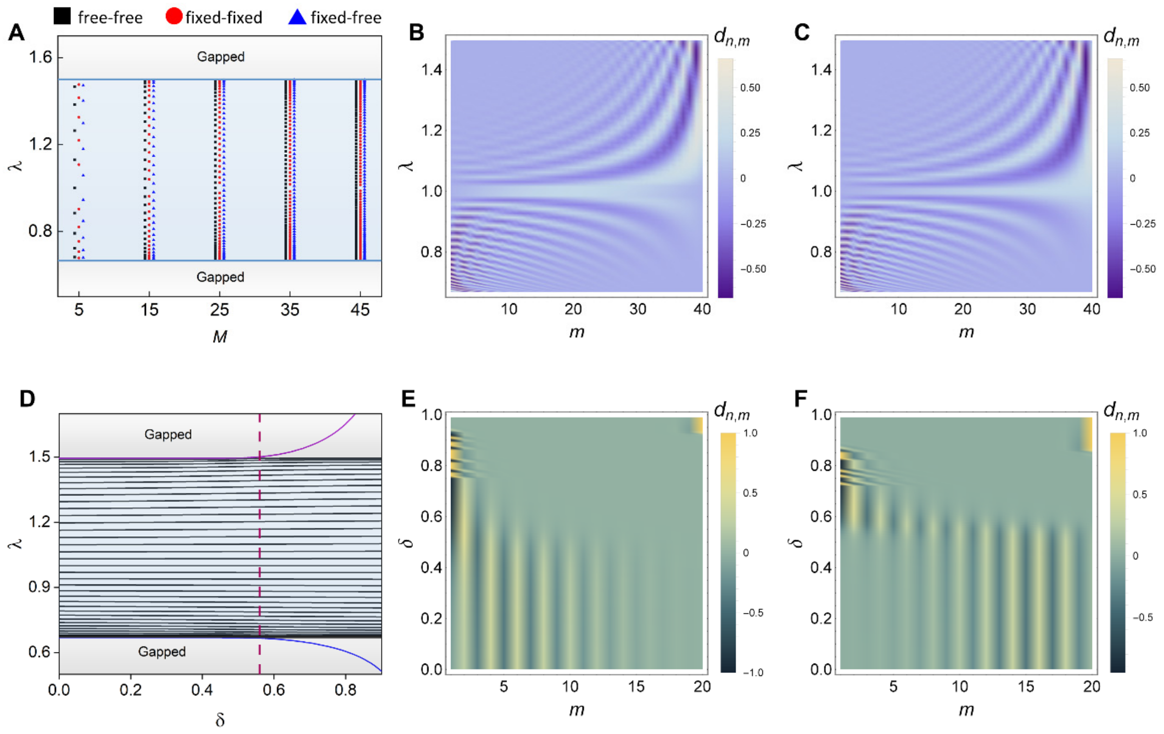

Fig. S8.

Non-Hermitian topological robustness to boundary conditions and local defects. (**A**) The OBC eigenvalues captured from a finite model with different sizes, while the OBC is either free-free (black squares), fixed-fixed (red circles) or fixed-free (blue triangles). (**B** and **C**) Displacement distributions under the fixed-fixed (B) and fixed-free (C) OBCs. (**D**) Evolution of the OBC spectrum with respect to the defect strength, δ, which is defined as the reduction of the horizontal stiffness at bottom end, $m = 1$. Black lines represent the bulk spectrum, while purple and blue lines denote the highest (with the largest eigenvalue) and lowest (with the lowest eigenvalue) branches, respectively. (**E** and **F**) Displacement distributions with respect to δ for the lowest (E) and highest (F) branches, respectively. Stiffnesses are $(k_1, k_2) = (0.01, 0.03)$ for all cases, with the size $M = 40$ for (B) and (C) and $M = 20$ for (D) to (F).

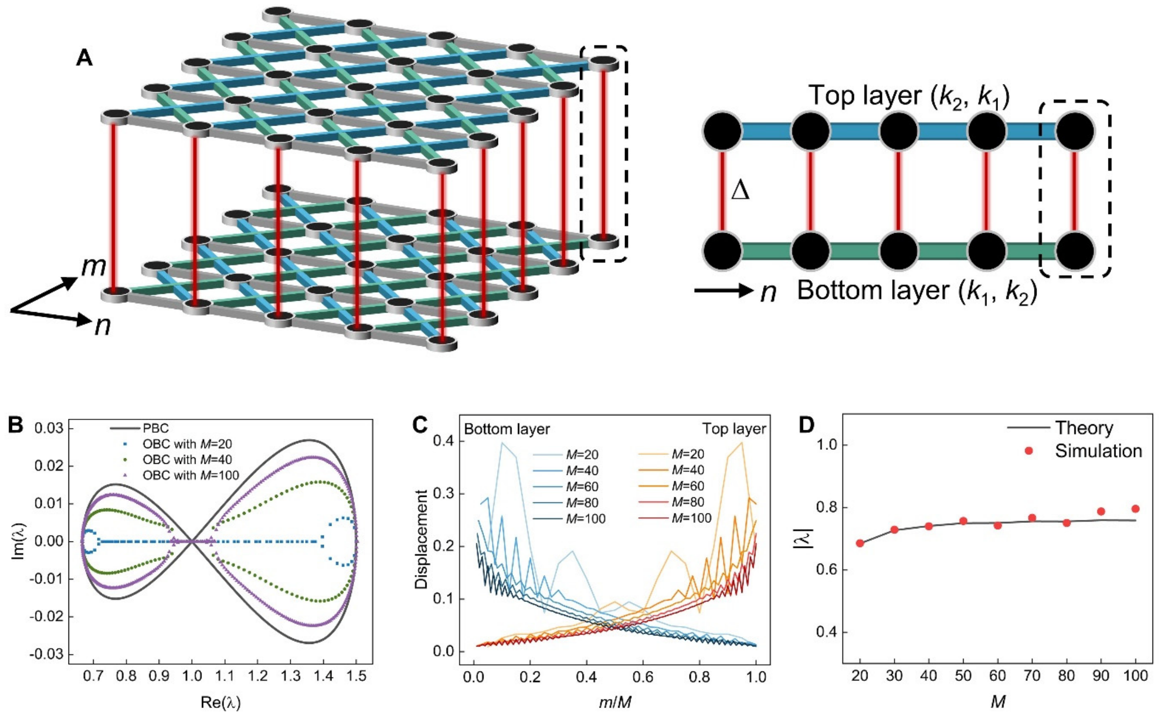

Fig. S9.

Critical non-Hermitian skin effect in a bilayer short-range lattice. (**A**) Schematic of a bilayer short-range lattice with switched diagonal stiffnesses. (**B**) PBC spectrum (black line) and OBC spectra with different system sizes (colored solid dots). (**C**) Normalized displacement distributions of the OBC eigenstates with maximum imaginary decay factors, sharing an identical decay rate of the whole lattice. (**D**) Theoretical and simulation results of the moduli of the decay factors in (C) for different system sizes. The stiffnesses are $(k_1, k_2) = (0.01, 0.03)$ and $\Delta = 0.001$.

Movie S1.

Design strategy. We use the dot-bar model to pictorially illustrate our idea. Black dots and gray bars denote the nodes and struts of the static metamaterial respectively. As comparison, the dynamical mass-spring model with the mass and spring depicted by the black sphere and helix respectively, are constructed.

Movie S2.

Experimental verification of the non-Hermitian skin effect. We experimentally verify the NHSE in our static metamaterials inspired from the Rayleigh-Ritz method. Prescribed displacement boundary conditions are applied at both the left and right boundaries, and the corresponding collective decay mode of the middle column can be excited, hence confirming the NHSE.

Movie S3.

Experimental verification for unidirectional load transfer as a dynamic signature of the skin effect. We experimentally verify the unidirectional transfer of an applied concentrated displacement-controlled load in such metamaterials. Here, the transfer of applied load in reciprocal lattices is almost symmetric about the central axis, while it is unidirectionally localized at a certain boundary in non-reciprocal metamaterials owing to the NHSE.

REFERENCES AND NOTES

1. Y. Ashida, Z. Gong, M. Ueda, Non-Hermitian physics. *Adv. Phys.* **69**, 249–435 (2021).

2. C. M. Bender, Making sense of non-Hermitian Hamiltonians. *Rep. Prog. Phys.* **70**, 947–1018 (2007).

3. A. Guo, G. J. Salamo, D. Duchesne, R. Morandotti, M. Volatier-Ravat, V. Aimez, G. A. Siviloglou, D. N. Christodoulides, Observation of PT-symmetry breaking in complex optical potentials. *Phys. Rev. Lett.* **103**, 093902 (2009).

4. K. G. Makris, R. El-Ganainy, D. N. Christodoulides, Z. H. Musslimani, Beam dynamics in PT symmetric optical lattices. *Phys. Rev. Lett.* **100**, 103904 (2008).

5. L. Feng, Z. J. Wong, R.-M. Ma, Y. Wang, X. Zhang, Single-mode laser by parity-time symmetry breaking. *Science* **346**, 972–975 (2014).

6. Z. Lin, H. Ramezani, T. Eichelkraut, T. Kottos, H. Cao, D. N. Christodoulides, Unidirectional invisibility induced by PT-symmetric periodic structures. *Phys. Rev. Lett.* **106**, 213901 (2011).

7. A. Ghatak, M. Brandenbourger, J. van Wezel, C. Coulais, Observation of non-Hermitian topology and its bulk–Edge correspondence in an active mechanical metamaterial. *Proc. Natl. Acad. Sci. U.S.A.* **117**, 29561–29568 (2020).

8. Q. Liang, D. Xie, Z. Dong, H. Li, H. Li, B. Gadway, W. Yi, B. Yan, Dynamic signatures of non-Hermitian skin effect and topology in ultracold atoms. *Phys. Rev. Lett.* **129**, 070401 (2022).

9. R. Fleury, D. Sounas, A. Alù, An invisible acoustic sensor based on parity-time symmetry. *Nat. Commun.* **6**, 5905 (2015).

10. Y. Li, Y.-G. Peng, L. Han, M.-A. Miri, W. Li, M. Xiao, X.-F. Zhu, J. Zhao, A. Alù, S. Fan, C.-W. Qiu, Anti-parity-time symmetry in diffusive systems. *Science* **364**, 170–173 (2019).

11. Y. Wu, W. Liu, J. Geng, X. Song, X. Ye, C.-K. Duan, X. Rong, J. Du, Observation of parity-time symmetry breaking in a single-spin system. *Science* **364**, 878–880 (2019).

12. C. Lv, R. Zhang, Z. Zhai, Q. Zhou, Curving the space by non-Hermiticity. *Nat. Commun.* **13**, 2184 (2022).

13. H. Fan, Hermitian and non-Hermitian topological edge states in one-dimensional perturbative elastic metamaterials. *Mech. Syst. Signal Process.* **169**, 108774 (2022).

14. R. Süsstrunk, S. D. Huber, Observation of phononic helical edge states in a mechanical topological insulator. *Science* **349**, 47–50 (2015).

15. K. L. Tsakmakidis, L. Shen, S. A. Schulz, X. Zheng, J. Upham, X. Deng, H. Altug, A. F. Vakakis, R. W. Boyd, Breaking Lorentz reciprocity to overcome the time-bandwidth limit in physics and engineering. *Science* **356**, 1260–1264 (2017).

16. Y. Chen, X. Li, C. Scheibner, V. Vitelli, G. Huang, Realization of active metamaterials with odd micropolar elasticity. *Nat. Commun.* **12**, 5935 (2021).

17. H. Nassar, B. Yousefzadeh, R. Fleury, M. Ruzzene, A. Alù, C. Daraio, A. N. Norris, G. Huang, M. R. Haberman, Nonreciprocity in acoustic and elastic materials. *Nat. Rev. Mater.* **5**, 667–685 (2020).

18. M. Brandenbourger, X. Locsin, E. Lerner, C. Coulais, Non-reciprocal robotic metamaterials. *Nat. Commun.* **10**, 4608 (2019).

19. L. Feng, M. Ayache, J. Huang, Y.-L. Xu, M.-H. Lu, Y.-F. Chen, Y. Fainman, A. Scherer, Nonreciprocal light propagation in a silicon photonic circuit. *Science* **333**, 729–733 (2011).

20. Z. Chen, Y. Peng, H. Li, J. Liu, Y. Ding, B. Liang, X.-F. Zhu, Y. Lu, J. Cheng, A. Alù, Efficient nonreciprocal mode transitions in spatiotemporally modulated acoustic metamaterials. *Sci. Adv.* **7**, eabj1198 (2021).

21. C. Coulais, D. Sounas, A. Alù, Static non-reciprocity in mechanical metamaterials. *Nature* **542**, 461–464 (2017).

22. M. Fruchart, R. Hanai, P. B. Littlewood, V. Vitelli, Non-reciprocal phase transitions. *Nature* **592**, 363–369 (2021).

23. K. Takata, M. Notomi, Photonic topological insulating phase induced solely by gain and loss. *Phys. Rev. Lett.* **121**, 213902 (2018).

24. M. Pan, H. Zhao, P. Miao, S. Longhi, L. Feng, Photonic zero mode in a non-Hermitian photonic lattice. *Nat. Commun.* **9**, 1308 (2018).

25. Z. Gong, Y. Ashida, K. Kawabata, K. Takasan, S. Higashikawa, M. Ueda, Topological phases of non-Hermitian systems. *Phys. Rev. X.* **8**, 031079 (2018).

26. N. Okuma, K. Kawabata, K. Shiozaki, M. Sato, Topological origin of non-Hermitian skin effects. *Phys. Rev. Lett.* **124**, 086801 (2020).

27. K. Wang, A. Dutt, K. Y. Yang, C. C. Wojcik, J. Vučković, S. Fan, Generating arbitrary topological windings of a non-Hermitian band. *Science* **371**, 1240–1245 (2021).

28. K. Ding, C. Fang, G. Ma, Non-Hermitian topology and exceptional-point geometries. *Nat. Rev. Phys.* **4**, 745–760 (2022).

29. Q. Zhang, Y. Li, H. Sun, X. Liu, L. Zhao, X. Feng, X. Fan, C. Qiu, Observation of acoustic non-Hermitian Bloch braids and associated topological phase transitions. *Phys. Rev. Lett.* **130**, 017201 (2023).

30. S. Yao, Z. Wang, Edge states and topological invariants of non-Hermitian systems. *Phys. Rev. Lett.* **121**, 086803 (2018).

31. C. H. Lee, R. Thomale, Anatomy of skin modes and topology in non-Hermitian systems. *Phys. Rev. B* **99**, 201103 (2019).

32. K. Kawabata, M. Sato, K. Shiozaki, Higher-order non-Hermitian skin effect. *Phys. Rev. B.* **102**, 205118 (2020).

33. T. Hofmann, T. Helbig, F. Schindler, N. Salgo, M. Brzezinska, M. Greiter, T. Kiessling, D. Wolf, A. Vollhardt, A. Kabaši, C. H. Lee, A. Bilušic, R. Thomale, T. Neupert, Reciprocal skin effect and its realization in a topolectrical circuit. *Phys. Rev. Res.* **2**, 023265 (2020).

34. X. Zhang, Y. Tian, J.-H. Jiang, M.-H. Lu, Y.-F. Chen, Observation of higher-order non-Hermitian skin effect. *Nat. Commun.* **12**, 5377 (2021).

35. E. G. Karpov, Structural metamaterials with Saint-Venant edge effect reversal. *Acta Mater.* **123**, 245–254 (2017).

36. C. O. Horgan, J. G. Simmonds, Saint-Venant end effects in composite structures. *Compos. Eng.* **4**, 279–286 (1994).

37. N. Hatano, D. R. Nelson, Localization transitions in non-Hermitian quantum mechanics. *Phys. Rev. Lett.* **77**, 570–573 (1996).

38. N. Hatano, D. R. Nelson, Vortex pinning and non-Hermitian quantum mechanics. *Phys. Rev. B* **56**, 8651–8673 (1997).

39. H. Chen, H. Zhang, Q. Wu, Y. Huang, H. Nguyen, E. Prodan, X. Zhou, G. Huang, Creating synthetic spaces for higher-order topological sound transport. *Nat. Commun.* **12**, 5028 (2021).

40. E. Lustig, S. Weimann, Y. Plotnik, Y. Lumer, M. A. Bandres, A. Szameit, M. Segev, Photonic topological insulator in synthetic dimensions. *Nature* **567**, 356–360 (2019).

41. M. C. Rechtsman, J. M. Zeuner, Y. Plotnik, Y. Lumer, D. Podolsky, F. Dreisow, S. Nolte, M. Segev, A. Szameit, Photonic Floquet topological insulators. *Nature* **496**, 196–200 (2013).

42. A. Wang, Y. Zhou, C. Q. Chen, Topological mechanics beyond wave dynamics. *J. Mech. Phys. Solids* **173**, 105197 (2023).

43. N. G. Stephen, Transfer matrix analysis of the elastostatics of one-dimensional repetitive structures. *Proc. R. Soc. A* **462**, 2245–2270 (2006).

44. R. Süsstrunk, S. D. Huber, Classification of topological phonons in linear mechanical metamaterials. *Proc. Natl. Acad. Sci. U.S.A.* **113**, E4767 (2016).

45. E. J. Bergholtz, J. C. Budich, F. K. Kunst, Exceptional topology of non-Hermitian systems. *Rev. Mod. Phys.* **93**, 015005 (2021).

46. F. Song, S. Yao, Z. Wang, Non-Hermitian topological invariants in real space. *Phys. Rev. Lett.* **123**, 246801 (2019).

47. S. Weidemann, M. Kremer, T. Helbig, T. Hofmann, A. Stegmaier, M. Greiter, R. Thomale, A. Szameit, Topological funneling of light. *Science* **368**, 311–314 (2020).

48. W. Wang, X. Wang, G. Ma, Non-Hermitian morphing of topological modes. *Nature* **608**, 50–55 (2022).

49. L. Li, C. H. Lee, S. Mu, J. Gong, Critical non-Hermitian skin effect. *Nat. Commun.* **11**, 5491 (2020).

50. K. Yokomizo, S. Murakami, Scaling rule for the critical non-Hermitian skin effect. *Phys. Rev. B* **104**, 165117 (2021).

51. S. Longhi, D. Gatti, G. D. Valle, Robust light transport in non-Hermitian photonic lattices. *Sci. Rep.* **5**, 13376 (2015).

52. L. Zhang, Y. Yang, Y. Ge, Y.-J. Guan, Q. Chen, Q. Yan, F. Chen, R. Xi, Y. Li, D. Jia, S.-Q. Yuan, H.-X. Sun, H. Chen, B. Zhang, Acoustic non-Hermitian skin effect from twisted winding topology. *Nat. Commun.* **12**, 6297 (2021).

53. C. Coulais, R. Fleury, J. van Wezel, Topology and broken Hermiticity. *Nat. Phys.* **17**, 9–13 (2021).

54. Y. Zhou, Y. Zhang, C. Q. Chen, Amplitude-dependent boundary modes in topological mechanical lattices. *J. Mech. Phys. Solids* **153**, 104482 (2021).

55. Y. Zhang, B. Li, Q. S. Zheng, G. M. Genin, C. Q. Chen, Programmable and robust static topological solitons in mechanical metamaterials. *Nat. Commun.* **10**, 5605 (2019).

56. Y. Chen, Q. Zhang, Y. Zhang, B. Xia, X. Liu, X. Zhou, C. Chen, G. Hu, Research progress of elastic topological materials. *Adv. Mech.* **51**, 189–256 (2021).

57. R. Okugawa, R. Takahashi, K. Yokomizo, Non-Hermitian band topology with generalized inversion symmetry. *Phys. Rev. B* **103**, 205205 (2021).

58. K. Kawabata, N. Okuma, M. Sato, Non-Bloch band theory of non-Hermitian Hamiltonians in the symplectic class. *Phys. Rev. B.* **101**, 195147 (2020).

59. J. T. Klein, E. G. Karpov, Exact analytical solutions in two dimensional plate-like mechanical metamaterials: State of free deformation in a topological cylinder. *Int. J. Mech. Sci.* **167**, 105292 (2020).

60. F. G. Scholtz, H. B. Geyer, F. J. W. Hahne, Quasi-Hermitian operators in quantum mechanics and the variational principle. *Ann. Phys.* **213**, 74–101 (1992).

61. V. M. Martinez Alvarez, J. E. Barrios Vargas, L. E. F. Foa Torres, Non-Hermitian robust edge states in one dimension: Anomalous localization and eigenspace condensation at exceptional points. *Phys. Rev. B* **97**, 121401 (2018).

62. R. Fleury, Non-local oddities. *Nat. Phys.* **17**, 766–767 (2021).

63. H. Chen, H. Nassar, G. L. Huang, A study of topological effects in 1D and 2D mechanical lattices. *J. Mech. Phys. Solids* **117**, 22–36 (2018).

64. Z. Yang, K. Zhang, C. Fang, J. Hu, Non-Hermitian bulk-boundary correspondence and auxiliary generalized Brillouin zone theory. *Phys. Rev. Lett.* **125**, 226402 (2020).

65. S. Yao, F. Song, Z. Wang, Non-Hermitian Chern bands. *Phys. Rev. Lett.* **121**, 136802 (2018).

66. K. Yokomizo, S. Murakami, Non-Bloch band theory of non-Hermitian systems. *Phys. Rev. Lett.* **123**, 066404 (2019).

67. K. Kawabata, K. Shiozaki, M. Ueda, M. Sato, Symmetry and topology in non-Hermitian physics. *Phys. Rev. X* **9**, 041015 (2019).

68. Y. Yi, Z. Yang, Non-Hermitian skin modes induced by on-site dissipations and chiral tunneling effect. *Phys. Rev. Lett.* **125**, 186802 (2020).

69. L. Li, C. H. Lee, J. Gong, Impurity induced scale-free localization. *Commun. Phys.* **4**, 42 (2021).

70. R. Arouca, C. H. Lee, C. Morais Smith, Unconventional scaling at non-Hermitian critical points. *Phys. Rev. B* **102**, 245145 (2020).

71. S. M. Rafi-Ul-Islam, Z. B. Siu, H. Sahin, C. H. Lee, M. B. A. Jalil, Critical hybridization of skin modes in coupled non-Hermitian chains. *Phys. Rev. Res.* **4**, 013243 (2022).

72. S. Longhi, Non-Hermitian gauged topological laser arrays. *Ann. Phys.* **530**, 1800023 (2018).

73. W. Zhu, W. X. Teo, L. Li, J. Gong, Delocalization of topological edge states. *Phys. Rev. B* **103**, 195414 (2021).

74. K. Zhang, Z. Yang, C. Fang, Universal non-Hermitian skin effect in two and higher dimensions. *Nat. Commun.* **13**, 2496 (2022).

75. C. H. Lee, L. Li, J. Gong, Hybrid higher-order skin-topological modes in nonreciprocal systems. *Phys. Rev. Lett.* **123**, 016805 (2019).
